# Sucrose addition directionally enhances bacterial community convergence and network stability of the shrimp culture system

**DOI:** 10.1038/s41522-022-00288-x

**Published:** 2022-04-11

**Authors:** Haipeng Guo, Pengsheng Dong, Fan Gao, Lei Huang, Sipeng Wang, Ruoyu Wang, Mengchen Yan, Demin Zhang

**Affiliations:** 1 State key laboratory for managing biotic and chemical threats to the quality and safety of agro-products, Ningbo University, Ningbo, 315211 China; 2 School of Marine Sciences, Ningbo University, Ningbo, 315211 China

**Keywords:** Microbial ecology, Water microbiology

## Abstract

Sucrose is an effective carbon source for creating more reliable and environmentally friendly conditions for shrimp growth by regulating bacteria in biofloc-based culture systems. However, the influence of sucrose addition on the interaction, co-occurrence networks, and assembly mechanisms of bacterial communities in biofloc-based culture systems remains largely unknown. Here, we comprehensively investigated the effects of sucrose addition on bacterial communities in three habitats (water, bioflocs, and gut). The bacterial community structures and compositions of these three habitats became more similar in groups with sucrose addition, compared with those in controls. More than 50% gut bacterial communities were mainly derived from water and biofloc communities in the sucrose addition groups, but only about 33% bacterial communities migrated from water and biofloc to the gut in the control culture system. Sucrose addition accordantly enriched core taxa belonging to the phylum Actinobacteria and the families Rhodobacteraceae and Flavobacteriaceae in water, biofloc, and gut habitats. These core taxa were important for maintaining bacterial network stability in the sucrose addition culture systems and some were identified as keystone taxa for improving shrimp growth. Furthermore, after sucrose addition, gut bacterial community assembly from water and biofloc was dominated by the heterogeneous select with the ratios of 55–91% and 67–83%, respectively, indicating that sucrose addition can directionally shape the bacterial assembly of the shrimp culture system. These results provide a basis for selectively regulating certain beneficial taxa to improve shrimp growth in culture systems.

## Introduction

Pacific white shrimp (*Penaeus vannamei*) is regarded as the most important farmed single crustacean species, accounting for 53% total farmed crustacean production due to the development of intensive shrimp aquaculture^1^. However, shrimp intensification has caused increasingly frequent disease outbreaks, resulting in great economic loss^2^. Biofloc technology (BFT), which works via supplementing carbon sources to achieve a carbon/nitrogen (C/N) ratio of more than 10 in the rearing water, is widely proposed to reduce disease outbreaks of shrimp in intensive aquaculture production systems^3^. This aquaculture system is beneficial for maintaining environmental sustainability and ecosystem stability, which greatly rely on the contribution of microbial communities, especially the heterotrophic bacteria stimulated by the high C/N ratio in the rearing water^4^.

Microbial communities play a restraining role in the productivity and sustainability of aquaculture ecosystems because they are key drivers of many ecosystem processes, including nutrient cycling, organic matter decomposition, and symbiotic and pathogenic interactions with aquatic animals^4,5^. In biofloc-based systems, these ecosystem processes may be strengthened by a large accumulation of heterotrophic bacteria in ponds, which not only efficiently transfers ammonium or organic nitrogenous waste into bacterial protein, but also forms a conglomerate of microbes for shrimp ingestion^5^. The improved environmental conditions in turn have positive effects on the stability of environmental microbiota, primarily by enriching potential probiotic species and depleting certain pathogens, thus providing an advantageous environment for reared shrimp^6^. Moreover, beneficial bacteria in water and bioflocs can be transferred to the shrimp gut through the oral route, remarkably affecting the shrimp gut microbiota^3,7^. Previous research revealed that the shrimp gut microbiota is closely associated with the environmental microbiota, especially in biofloc-based culture systems^8,9^. However, a deeper understanding of how environmental microbiota affect gut microbiota and the interactions between shrimp intestine and environmental microbiota is lacking. This is essential information for constructing microbial ecological strategies to manipulate gut microbiota and maintain the healthy growth of shrimp^6,10^.

Interspecies interaction is crucial for ecosystem services^11^. In recent years, microbial co-occurrence network analysis has been used to reveal microbe–microbe interactions in communities^11^. Network properties can be used to predict the stability of microbial networks. Previous studies indicate that destabilizing microbial communities are responsible for the occurrence of disease in plants^12^, animals^13^, and humans^14^. In shrimp, our findings demonstrate that bacterial communities in healthy shrimp or low-temperature tolerant shrimp species are more stable than those in diseased shrimp or low-temperature-sensitive species^15,16^, while dysbiosis generally leaves shrimp vulnerable to pathogen infection and environmental stress^17^. Biofloc culture systems represent a competitive environment with a low substrate supply per cell due to high biomass^6^. The addition of a carbon source might alter the stability of the bacterial community, which in turn has implications for shrimp growth performance. In addition to stability, network analysis is also applied for statistically identifying keystone taxa that generally exert a considerable effect on microbial community structure and functioning irrespective of their abundance^18^. A number of studies have reported that the impact of bioflocs on the shrimp culture system microbiome is facilitated via keystone taxa^19,20^. However, whether bacterial networks differ between non-biofloc and biofloc-based culture systems has not been investigated. Moreover, the ecological process shaping microbial diversity and structure is critical for determining links between community stability and ecosystem functions^21,22^, yet the effects of carbon source addition on the assembly mechanism of bacterial communities in shrimp culture systems remain poorly understood.

In this study, we systematically analyzed differences in bacterial community structure and composition among water, biofloc, and gut communities in control and sucrose addition culture systems. We then contrasted the bacterial networks in the different culture systems to reveal: (1) whether network stability is changed by sucrose addition using modularity, network cohesion, and robustness, and (2) which taxa act as keystone taxa, and their associations with shrimp growth performance. We also evaluated which ecological processes shape the microbial community structures in different culture systems. In addition, core taxa and specific taxa were defined to further understand the differences in bacterial community composition, stability, and assembly mechanisms in these different culture systems.

## Results

### Effects of sucrose addition on water quality parameters, biofloc formation, and shrimp growth performance

Water quality parameters such as Chla, NO_3_-N, NH_4_-N, NO_2_-N, and PO_4_-P showed a decreasing trend in CN10 and CN15 groups, compared to those in the CK group (Supplementary Fig. 1). This was especially apparent for contents of NO_3_-N, NO_2_-N, and PO_4_-P, which were up to 16.0, 33.0, and 1.23 mg L^−1^ in the CK group, while they were reduced to 0.08, 0.07, and 0.09 mg L^−1^ in the CN15 group, respectively. Sucrose addition significantly increased (Student’s t test *p* < 0.05) COD contents by 40.4% and 49.4% in CN10 and CN15 groups, respectively, compared with that in the CK group. Bioflocs were rarely formed in the CK group, while they were obviously formed after 10 d of sucrose addition; biofloc volumes were 14.1 and 31.8 mL L^−1^ at the end of experiment in CN10 and CN15 groups, respectively (Supplementary Fig. 1). Sucrose addition markedly improved (Student’s *t* test *p* < 0.05) the growth performance of shrimps, especially in the CN15 group; SRG, SR, and total yield were 4.8-, 2.0- and 3.6-fold higher than those in the CK group, respectively (Supplementary Fig. 1).

### Effects of sucrose addition on water, biofloc, and gut bacterial community composition

**Fig. 1:**
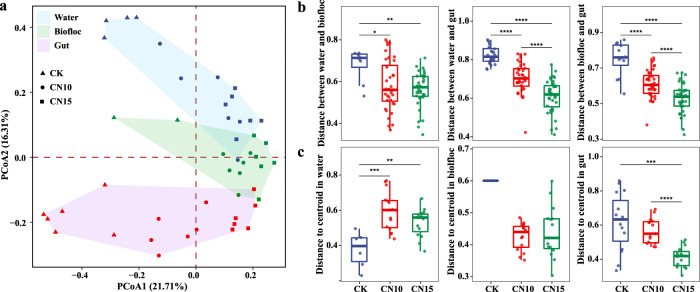
Effects of sucrose addition on the bacterial community structure of rearing water, biofloc and gut in the shrimp culture systems. **a** Principal coordinate analysis (PCoA) plot based on the Bray-Curtis dissimilarity shows the dissimilarities of bacterial community between control and sucrose addition groups in rearing water, biofloc, and gut samples. **b** The pairwise dissimilarities of bacterial community of rearing water, biofloc and gut in control and sucrose addition culture systems. **c** The dissimilarities of bacterial communities within group of rearing water, biofloc and gut samples in control and sucrose addition culture systems. CK: without sucrose addition; CN10: the C/N ratio of about 10:1 obtained by sucrose addition; CN15: the C/N ratio of about 15:1 obtained by sucrose addition. Each sample had six replicates (*n* = 6), except the water samples (*n* = 4) and biofloc samples (*n* = 2) in control group. The boxes represent the median and interquartile range, and whiskers range from minimum to maximum values. Different asterisks indicate a significant difference at *p < 0.05, **p < 0.01, ***p < 0.001 and ****p < 0.0001 in (**b**) and (**c**) based on Student’s *t* test.

Bacterial richness, evenness, and phylogenetic diversity showed no significant differences (Student’s *t* test *p* > 0.05) between control and sucrose addition groups in water; meanwhile, they were markedly increased (Student’s *t* test *p* < 0.05) by sucrose addition in biofloc and gut (Supplementary Fig. 2a). Variations in these alpha-diversity indexes within sucrose addition groups were either increased or slightly decreased in water and biofloc, compared with those of the control. By contrast, smaller variations within CN10 and CN15 groups were detected in the gut, compared with those of CK (Supplementary Fig. 2b). Although PCoA1 and PCoA2 together contribute only 38% variances, significant differences (ANOSIM and PERMANOVA *p* < 0.05) in bacterial community composition for water, biofloc, and gut were found via PCoA analysis after sucrose addition. These differences were increased with greater sucrose addition according to both ANOSIM and PERMANOVA analyses (Fig. 1a and Table 1). Sucrose addition also significantly changed (ANOSIM and PERMANOVA *p* < 0.05) the bacterial similarity among water, biofloc, and gut, which became more similar along the PCoA1 axis (Fig. 1a and Table 1). This trend was also proved by significantly shortened distances between water and biofloc, water and gut, and biofloc and gut (Fig. 1b). In addition, sucrose addition significantly increased (Student’s *t* test *p* < 0.01) the fluctuation of within-group bacterial community in water, demonstrated by increased centroid distance, while it made the within-group bacterial community in gut more similar (Fig. 1c).

**Table 1:** ANOSIM and PERMANOVA analysis between different groups.

Group	Process	ANOSIM		PERMANOVA		
		*R*	*P*_adj_BH	*F* value	Variation (*R*^2^)	*P*_adj_BH
CK	Water vs Biofloc	0.93	0.07	4.22	0.51	0.067
	Water vs Gut	0.80	0.007	6.95	0.47	0.008
	Biofloc vs Gut	0.47	0.154	2.33	0.28	0.045
CN10	Water vs Biofloc	0.37	0.007	2.72	0.21	0.008
	Water vs Gut	0.69	0.007	3.75	0.27	0.008
	Biofloc vs Gut	0.65	0.007	3.94	0.28	0.008
CN15	Water vs Biofloc	0.47	0.008	2.98	0.23	0.008
	Water vs Gut	0.73	0.007	4.87	0.33	0.013
	Biofloc vs Gut	0.66	0.007	4.18	0.29	0.008
Water	CK vs CN10	0.66	0.018	4.87	0.38	0.011
	CK vs CN15	0.98	0.008	7.67	0.49	0009
	CN10 vs CN15	0.40	0.007	2.24	0.18	0.011
Biofloc	CK vs CN10	0.90	0.036	3.80	0.39	0.039
	CK vs CN15	0.94	0.031	4.34	0.42	0.040
	CN10 vs CN15	0.66	0.007	3.63	0.27	0.008
Gut	CK vs CN10	0.61	0.007	3.90	0.28	0.008
	CK vs CN15	0.88	0.007	8.93	0.47	0.008
	CN10 vs CN15	0.71	0.007	4.45	0.31	0.008

ANOSIM and PERMANOVA analysis were based on Bray-Curtis dissimilarity for the comparison of bacterial community composition in the water, biofloc, and gut samples between control and treatment (permutation = 999). Each sample had six replicates (*n* = 6), except the water samples (*n* = 4) and biofloc samples (*n* = 2) in control group. *P* values adjusted by using the Benjamini–Hochberg algorithm <0.05 indicate significant differences.

The bacterial community mainly consisted of Alphaproteobacteria, Bacteroidetes, Actinobacteria, Deltaproteobacteria, Planctomycetes and Chloroflexi in both water and bioflocs, but taxa belonging to Deltaproteobacteria and Chloroflexi were replaced by bacteria from Tenericutes and Gammaproteobacteria in the gut (Supplementary Fig. 3a). Sucrose addition significantly enhanced (Student’s *t* test *p* < 0.05) the relative abundances of Actinobacteria in water, biofloc and gut, which were 5.4-, 4.2- and 8.0-fold higher in the CN15 group compared with that in the CK group, respectively (Supplementary Fig. 3b). In addition, sucrose addition also significantly increased (Student’s *t* test *p* < 0.05) the relative abundances of Alphaproteobacteria, Bacteroidetes and Saccharibacteria, and markedly reduced (Student’s *t*-test *p* < 0.05) the relative abundances of Firmicutes and Tenericutes in the gut (Supplementary Fig. 3b).

### Shared taxa between water, biofloc, and gut after sucrose addition

**Fig. 2:**
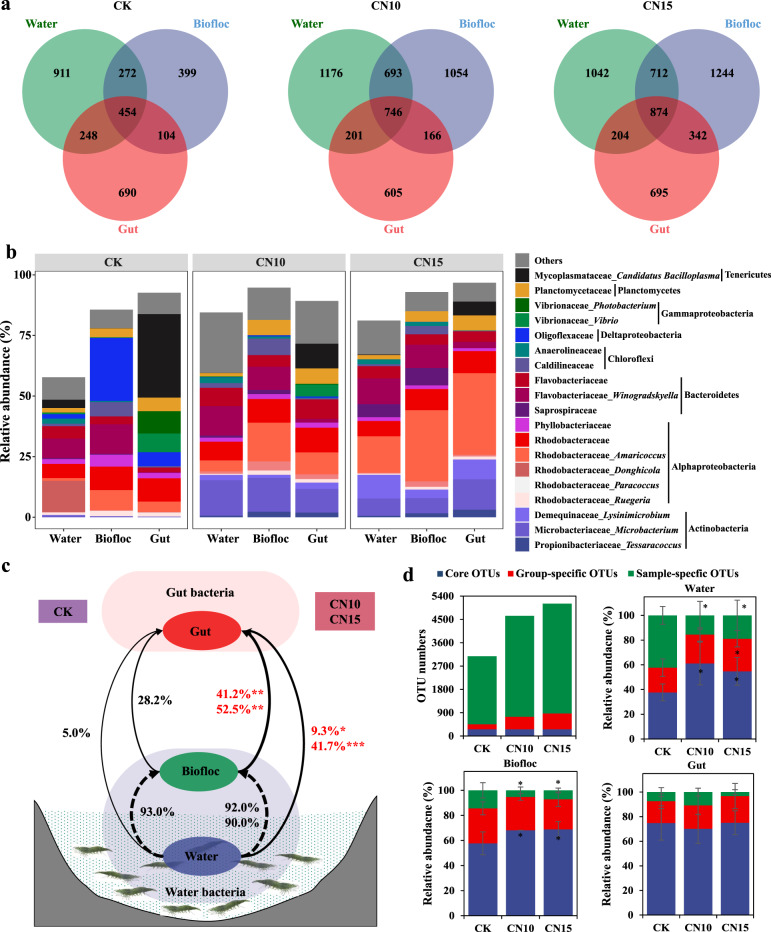
Effects of sucrose addition on the bacterial community compositions of rearing water, biofloc, and gut in the shrimp culture systems. **a** Venn analysis of bacterial communities at the OTU level among rearing water, biofloc, and gut in control and sucrose addition culture systems. **b** The relative abundances of bacterial taxa at genus level shared by rearing water, biofloc, and gut in each culture system. **c** The percentage contributions of bacterial sources for biofloc and gut samples in each culture system based on the SourceTracker analysis. **d** The numbers and relative abundances of core and specific OTUs in each culture system. CK: without sucrose addition; CN10: the C/N ratio of about 10:1 obtained by sucrose addition; CN15: the C/N ratio of about 15:1 obtained by sucrose addition. Each sample had six replicates (*n* = 6), except the water samples (*n* = 4) and biofloc samples (*n* = 2) in control group. Error bars show standard error in (**d**). Different asterisks indicate a significant difference at *p < 0.05, **p < 0.01, and ***p < 0.001 in (**c**) and (**d**) based on Student’s *t* test.

To understand the effects of sucrose addition on bacterial community composition in water, biofloc, and gut, we compared these communities at the OTU level using Venn analysis. The number of OTUs shared by the three habitats was increased by sucrose addition, with 454, 746, and 874 shared OTUs in CK, CN10, and CN15 groups, respectively (Fig. 2a). The dominant taxa among shared OTUs for each habitat were obviously distinct in the CK group and were mainly composed of Rhodobacteraceae and Flavobacteriaceae in water, Rhodobacteraceae, Flavobacteriaceae and Oligoflexaceae in biofloc, and Rhodobacteraceae, Vibrionaceae, and Mycoplasmataceae in gut, with total abundances of 57.8%, 85.7%, and 92.6%, respectively (Fig. 2b). However, the dominant taxa among shared OTUs for each habitat in CN10 and CN15 groups were more similar, mainly consisting of Rhodobacteraceae, Microbacteriaceae, Demequinaceae, and Flavobacteriaceae, with more than 80% total abundance in each habitat (Fig. 2b). These results indicate that sucrose addition may have reshaped the bacterial community of the shrimp culture system and increased bacterial migration from water or biofloc to the gut. To further verify this conclusion, a SourceTracker analysis was used to study the proportion of gut bacterial communities derived from water and biofloc (Fig. 2c). According to the source apportionment results, although the majority of biofloc bacterial community members (>90%) were derived from the water in both the control and sucrose addition groups, the sources of gut bacterial communities in control and sucrose addition groups were distinctly different (Student’s *t*-test *p* < 0.05). In the CK group, rare members of the gut bacterial community were derived from the water (5.0%) and biofloc (28.2%) bacterial communities, indicating that it was very hard for water bacteria to enter the gut in an unregulated culture system. In CN10 and CN15 groups, 9.3% and 41.7% of the gut bacterial community, respectively, was sourced from the water bacterial community, and 41.2% and 52.5% was sourced from the biofloc bacterial community, respectively, suggesting that most gut bacterial species could be tracked back to the water and biofloc after sucrose addition (Fig. 2c).

To clarify the influences of sucrose addition on the bacterial communities of the culture system, we divided the bacterial taxa into three types: core OTUs, group-specific OTUs, and sample-specific OTUs, according to their frequency of occurrence. A total of 258 OTUs shared by the culture systems of CK, CN10, and CN15 were identified as core OTUs, accounting for more than 50% abundance in almost all samples, except in water for the CK group (Fig. 2d). Although the relative abundances of core taxa in the gut of CK, CN10, and CN15 groups were up to 74.9%, 70.1%, and 75.1%, respectively, and were always higher than those in water and biofloc, there were no significant differences between control and sucrose addition groups in the gut (Fig. 2d). By contrast, the abundances of core taxa in water and biofloc were significantly increased (Student’s *t*-test *p* < *0.05*) by 45.0–62.0% and 18.0–19.3% after sucrose addition, respectively, compared with those of the control (Fig. 2d). The numbers of group-specific OTUs and sample-specific OTUs were enhanced by sucrose addition, but their abundances were either not significantly different or markedly decreased by sucrose addition, compared with those in the CK group, indicating that most specific OTUs in sucrose addition groups were rare taxa (Fig. 2d).

### Differentially abundant core and specific taxa in water, biofloc, and gut after sucrose addition

**Fig. 3:**
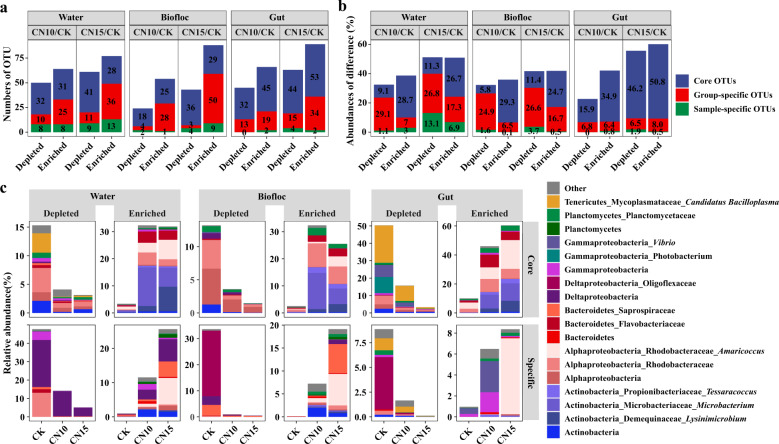
The numbers and relative abundances of discriminatory OTUs in rearing water, biofloc, and gut samples between control and sucrose addition culture systems. **a** Number of significantly discriminatory OTUs (*P*_FDR_ < 0.05) detected in rearing water, biofloc, and gut samples between control and sucrose addition groups. **b** The cumulative abundances of significantly enriched and depleted OTUs in rearing water, biofloc, and gut samples between control and sucrose addition groups. **c** The relative abundances and compositions of significantly enriched and depleted OTUs in rearing water, biofloc, and gut samples between control and sucrose addition groups. CK: without sucrose addition; CN10: the C/N ratio of about 10:1 obtained by sucrose addition; CN15: the C/N ratio of about 15:1 obtained by sucrose addition. Each sample had six replicates (*n* = 6), except the water samples (*n* = 4) and biofloc samples (*n* = 2) in control group.

To further identify the core and specific OTUs contributing to the divergence in bacterial community composition in the three habitats after sucrose addition, we determined differentially abundant OTUs based on DESeq2 analysis with OTU counts. Sucrose addition had a greater influence on specific OTUs than core OTUs in water and biofloc but had greater effects on core taxa than specific taxa in the gut, according to the numbers of differentially abundant OTUs and the size of these differences (Fig. 3a and b, Supplementary Table 1). Sucrose addition mainly induced accumulation of core OTUs in water and biofloc, but largely reduced the abundances of specific OTUs (especially the abundances of group-specific OTUs), which were reduced by 30.2–39.9%, and 26.5–30.3% in water and biofloc, respectively, compared with their respective controls (Fig. 3b). Notably, sucrose addition mainly changed the abundances of core OTUs in the gut, either depleting or enriching these OTUs (Fig. 3b and c). We combined the group-specific and sample-specific OTUs as specific OTUs in the following analysis due to the low numbers and abundances of differentially abundant sample-specific OTUs.

Although we observed different patterns of OTU abundance in water, biofloc, and gut in response to sucrose addition, the enriched core OTUs were highly similar and mainly belonged to Demequinaceae, Microbacteriaceae, and Propionibacteriaceae of Actinobacteria, Rhodobacteraceae of Alphaproteobacteria, Flavobacteriaceae of Bacteroidetes, and Planctomycetaceae of Planctomycetes. The core OTUs depleted by sucrose addition differed in the three habitats. The depleted taxa mainly belonged to Actinobacteria, Rhodobacteraceae, and Mycoplasmataceae in water, Actinobacteria, Alphaproteobacteria (almost half as Rhodobacteraceae), Deltaproteobacteria and Planctomycetes in biofloc, and Rhodobacteraceae, *Photobacterium*, *Vibrio* and *Candidatus Bacilloplasma* in gut (Fig. 3c). Specific OTUs depleted by sucrose addition mainly belonged to Rhodobacteraceae, Flavobacteriaceae, Deltaproteobacteria, and Gammaproteobacteria in water, Saprospiraceae and Oligoflexaceae in biofloc, and Oligoflexaceae and *Candidatus Bacilloplasma* in gut; while specific OTUs enriched by sucrose addition were primary associated with *Amaricoccus* (Rhodobacteraceae), Saprospiraceae and Actinobacteria (Fig. 3c). In addition, 71 significantly discriminatory OTUs between control and sucrose addition were shared by water, biofloc, and gut, including 48 core OTUs and 23 specific OTUs (Supplementary Figs. 4 and 5). It was remarkable that almost all shared specific OTUs were enriched by sucrose addition with log(fold change) of 1.8–12.6, and nine out of 23 belonged to Actinobacteria (especially in families Demequinaceae and Microbacteriace) (Fig. S5).

### Effects of sucrose addition on bacterial co-occurrence networks and stability of the culture system

To identify the effects of sucrose addition on microbe–microbe interactions and niche-sharing in the culture system, we constructed bacterial co-occurrence networks for CK, CN10, and CN15 groups using SparCC (Supplementary Fig. 6a). Sucrose addition increased the nodes but reduced the edges of the bacterial co-occurrence networks, resulting in low average connectivity and average clustering coefficients and a high average path length in CN10 and CN15 groups, compared with those in CK (Supplementary Table 2). The bacterial networks of sucrose addition groups had more nodes belonging to Bacteroidetes, Actinobacteria, and Deltaproteobacteria, and possessed fewer nodes assigned to Gammaproteobacteria than those of the control group (Supplementary Fig. 6a). The degrees and closeness centrality of all nodes, core nodes, and specific nodes were decreased, while the betweenness centrality of all nodes was increased by sucrose addition, especially in the CN10 group (Supplementary Fig. 6b). In addition, although the ratios of positive and negative interactions for all nodes in these bacterial networks were barely changed by sucrose addition, the relationships between core nodes and specific nodes were vastly different (Supplementary Table 3). Sucrose addition reduced positive interactions between core and core nodes but enhanced positive interactions between core and specific nodes and between specific and specific nodes (Supplementary Table 3), suggesting that core taxa may play an important role in recruiting specific taxa after sucrose addition to the culture system.

**Fig. 4:**
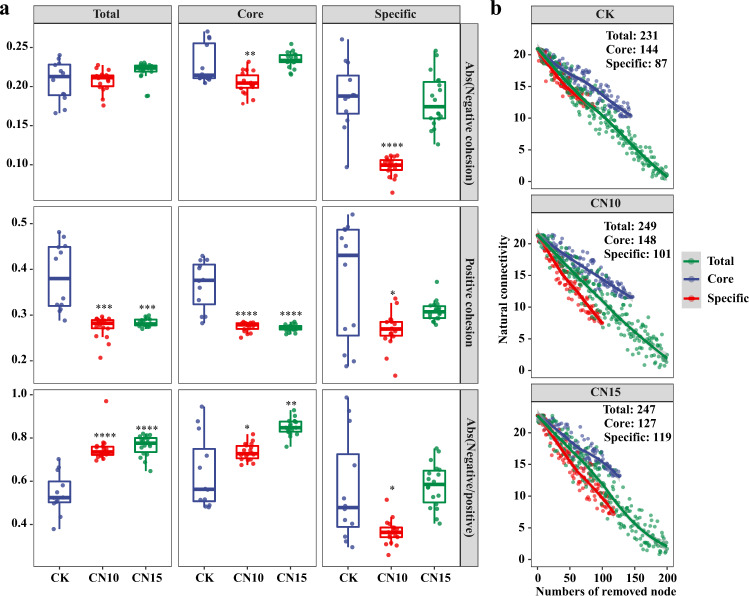
Effects of sucrose addition on the bacterial network properties of the culture systems. **a** Changes in absolute value of negative cohesion, positive cohesion, and absolute value of negative:positive cohesion ratio of total, core and specific bacterial communities in CK (*n* = 12), CN10 (*n* = 18), and CN15 (*n* = 18) groups. The boxes represent the median and interquartile range, and whiskers range from minimum to maximum values. Different asterisks indicate a significant difference at *p < 0.05, **p < 0.01, ***p < 0.001 and ****p < 0.0001 based on Student’s *t* test. **b** Changes in robustness (natural connectivity) of total, core and specific bacterial networks in CK (*n* = 12), CN10 (*n* = 18) and CN15 (*n* = 18) groups. CK: without sucrose addition; CN10: the C/N ratio of about 10:1 obtained by sucrose addition; CN15: the C/N ratio of about 15:1 obtained by sucrose addition.

Analysis of modularity, cohesion, and robustness indicated that the stability of bacterial networks was increased after sucrose addition. Sucrose addition increased the modularity of the bacterial community networks, with values of 1.665, 1.752, and 1.858 for CK, CN10, and CN15 groups, respectively (Supplementary Table 2), indicating that bacterial communities in the CN10 and CN15 groups were more compartmentalized than those in the CK group. Ratios of negative:positive cohesion of total taxa and core taxa were significantly increased following sucrose addition due to the remarkable reduction in positive cohesion (Fig. 4a; Student’s *t* test *p* < 0.001), indicating that negative associations rather than positive associations between bacteria in the total and core taxa dominated in the sucrose addition groups. The natural connectivity of the bacterial networks was always high when the core nodes were randomly removed, compared with when total nodes or specific nodes were randomly removed, in CK, CN10, and CN15 groups (Fig. 4b), indicating that core nodes were very important for maintaining the stability of bacterial networks in both the control group and sucrose addition groups. The natural connectivity dropped faster when specific nodes were randomly removed than when total nodes were randomly removed in CN10 and CN15 groups (Fig. 4b), suggesting that the roles of specific nodes in maintaining the stability of bacterial networks were strengthened by sucrose addition. In addition, the natural connectivity of the bacterial networks was always higher in sucrose addition groups than in the control group when total nodes or core nodes were randomly removed, while it was lower in CN10 and CN15 than in CK with increased removal of specific nodes (Supplementary Fig. 6c), indicating that the importance of core nodes in maintaining the robustness of bacterial networks was increased by sucrose addition.

### Roles of keystone taxa in linking water physicochemical properties and shrimp performances

**Fig. 5:**
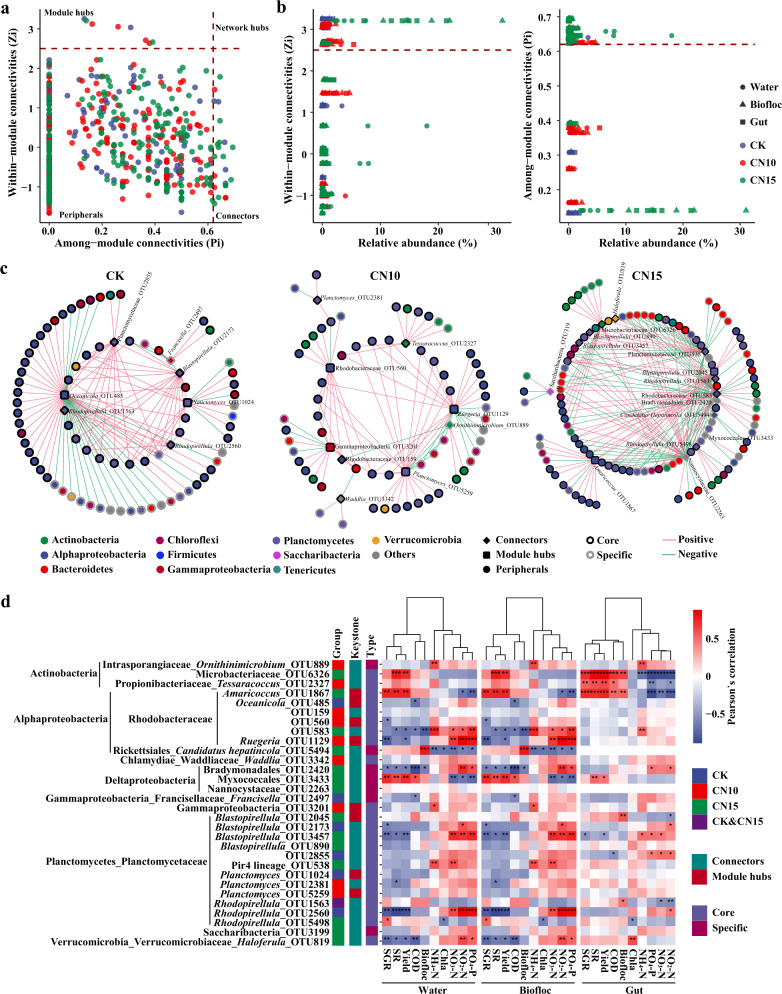
Identifying the keystone taxa of each bacterial networks and their associations with water quality and shrimp growth parameters. Keystone taxa (**a**) and their relative abundances (**b**) in bacterial communities of CK (*n* = 12), CN10 (*n* = 18) and CN15 (*n* = 18) groups. **c** The relationships of keystone OTUs and the OTUs they recruited in CK (*n* = 12), CN10 (*n* = 18) and CN15 (*n* = 18) groups. **d** Pearson’s correlation analysis between keystone OTUs, water quality parameters, biofloc volume and shrimp growth performance. NH_4_-N, ammonium-N; NO_2_-N, nitrite-N; NO_3_-N, nitrate- N; PO_4_-P, phosphate-P; COD, chemical oxygen demand; Chl-a, Chlorophyll a; SR, survival rate; SGR, specific growth rate; Yield, total yield of shrimp each pond. Different asterisks indicate a significant difference at *p < 0.05, **p < 0.01, and ***p < 0.001 in (**d**) based on Student’s *t* test. CK: without sucrose addition; CN10: the C/N ratio of about 10:1 obtained by sucrose addition; CN15: the C/N ratio of about 15:1 obtained by sucrose addition.

We calculated within module connectivity (Zi) and among-module connectivity (Pi) values of nodes in the networks to identify keystone taxa in the bacterial networks. Based on Zi and Pi values, all nodes were classified into four groups: peripherals, connectors, module hubs, and network hubs (Fig. 5a). The majority of nodes were peripherals, and no network hubs were identified in any network. There were two, four, and two module hubs, and five, five and 13 connectors in the bacterial networks of CK, CN10, and CN15 groups, respectively (Fig. 5a). The relative abundances of keystone OTUs in CK were generally <0.5%, while they were strongly enhanced in CN10 and CN15 groups, especially in the CN15 group (Fig. 5b and Supplementary Table 4). All module hubs were core taxa, while one out of five connectors in CK and CN10 groups and five out of 13 connectors in the CN15 group were specific taxa (Supplementary Table 4). The keystone OTUs in CK showed mainly positive associations with core OTUs assigned as Alphaproteobacteria and Planctomycetes and showed repulsive interactions with specific OTUs. By contrast, the keystone OTUs were mainly negatively associated with core OTUs from Alphaproteobacteria and Gammaproteobacteria and specific OTUs belonging to Bacteroidetes and Chloroflexi in the CN10 and CN15 groups. It is worth noting that keystone OTUs positively recruited core or specific OTUs belonging to Actinobacteria in CN10 and CN15 groups (Fig. 5c), implying that increased Actinobacteria abundance induced by sucrose addition may be beneficial for shrimp growth.

To further understand the roles of keystone OTUs in linking water quality and shrimp growth parameters, we calculated Pearson’s correlations between them (Fig. 5d). Two keystone OTUs belonging to Actinobacteria (OTU2327 and OTU6236) and one belonging to Alphaproteobacteria (OTU1867) were positively associated with SGR, SR, and total yield of shrimp in all three habitats, especially in the gut, where the positive associations were more significant (Fig. 5d; Student’s *t* test *p* < 0.01). Meanwhile, the relative abundances of these three keystone OTUs were significantly increased by sucrose addition in all three habitats (Supplementary Fig. 7). The keystone OTU2327 in the bacterial network of CN10 recruited eight OTUs, most showing a slight correlation with water quality change and shrimp growth. OTU1867 recruited 21 OTUs, including 18 core OTUs, which were mainly assigned as Rhodobacteraceae. Although the relative abundances of most core OTUs recruited by OTU1867 were not significantly increased by sucrose addition, most of them showed significantly positive associations with shrimp growth and biofloc formation and negative correlations with Chla, NH_4_-N, NO_2_-N, NO_3_-N and PO_4_-P (Supplementary Fig. 8). The keystone OTU6326 mainly induced aggregation of eight OTUs from Actinobacteria in water, biofloc, and gut: five specific OTUs and three core OTUs (Supplementary Fig. 9); these specific OTUs were also significantly and positively correlated with shrimp growth parameters, COD, and biofloc volume and negatively associated with water quality parameters such as Chla, NH_4_-N, NO_2_-N, NO_3_-N and PO_4_-P (Supplementary Fig. 8; Student’s *t* test *p* < 0.01).

Meanwhile, most keystone OTUs from Rhodobacteraceae and Planctomycetaceae were negatively associated with shrimp growth parameters and positively related to water quality parameters in water and biofloc, and the relative abundances of most of them were not significantly different between control and sucrose addition groups (Fig. 5d and Supplementary Fig. 7). OTU5494 from Rickettsiales showed significantly negative associations (Student’s *t* test *p* < 0.05) with water quality parameters and positive associations with biofloc volume in both water and biofloc (Fig. 5d).

### Effects of sucrose addition on the ecological processes of bacterial communities in the shrimp culture system

**Fig. 6:**
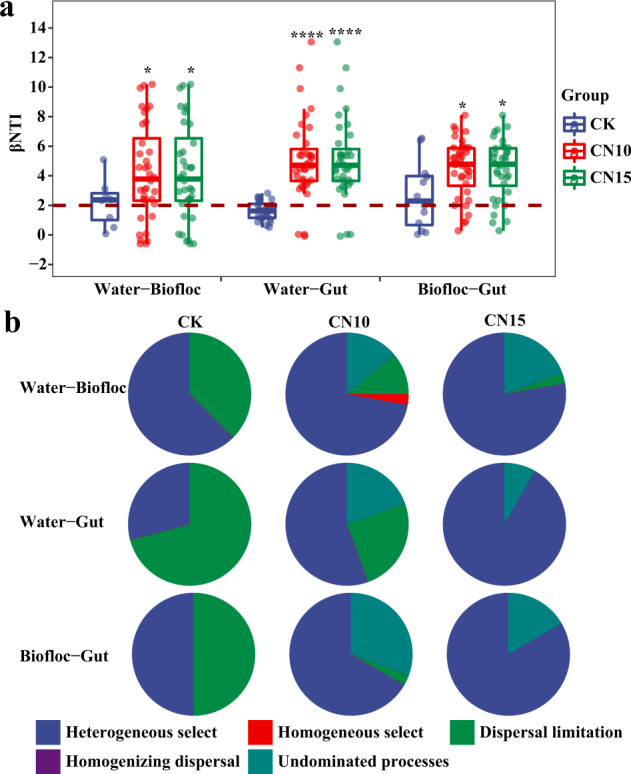
The ecological processes of bacteria from water to biofloc, water to gut, and biofloc to gut in control and sucrose addition groups. **a** The βNTI values. The boxes represent the median and interquartile range, and whiskers range from minimum to maximum values. Different asterisks indicate a significant difference at *p < 0.05, and ****p < 0.0001 based on Student’s *t* test. **b** The contributions of stochastic processes and deterministic process on the bacterial assembly. Each sample had six replicates (*n* = 6), except the water samples (*n* = 4) and biofloc samples (*n* = 2) in control group. CK: without sucrose addition; CN10: the C/N ratio of about 10:1 obtained by sucrose addition; CN15: the C/N ratio of about 15:1 obtained by sucrose addition.

There were significant differences in the assembly processes of bacterial communities from water to biofloc, water to gut, and biofloc to gut between control and sucrose addition groups based on the null model and Sloan neutral model (Fig. 6 and Supplementary Fig. 11). The majority of βNTI values were between −2 and 2 in the CK group, while they were significantly increased (Student’s *t* test *p* < 0.05) to more than 2 in the CN10 and CN15 groups (Fig. 6a), indicating that the influences of deterministic processes were enhanced by sucrose addition. The assembly processes of bacterial communities from water to biofloc were predominantly mediated by the deterministic process of heterogeneous selection in CK, and sucrose addition further increased this process by 15.6% and 24.4% in CN10 and CN15 groups, respectively, compared to that in CK (Fig. 6b). The assembly processes of bacterial communities from water to gut and from biofloc to gut were mainly governed by stochastic processes of dispersal limitation in CK (71% and 55%, respectively), whereas these processes changed to heterogeneous selection with values of 55% and 67% in CN10 and 91% and 83% in CN15, respectively (Fig. 6b). The Sloan neutral model indicated that the assembly patterns of the gut bacterial community only fit the neutral model when the bacterial community of water or biofloc in CK was used as the species pool. Although the assembly patterns of bacterial communities did not fit the neutral model in CN10 and CN15 groups, migration rates were all enhanced by sucrose addition (Supplementary Fig. 10).

Sucrose addition had a stronger influence on the deterministic process of specific taxa than core taxa. The deterministic process dominated the assembly of core taxa in CK, whether they were from water to biofloc and gut or from biofloc to gut, but these rules were broken by sucrose addition (Supplementary Fig. 11a and c). For the specific taxa, heterogeneous selection controlled the assembly processes from water to biofloc and from biolfloc to gut in all culture systems, and sucrose addition further enhanced this selective role (Supplementary Fig. 11b and d). Deterministic processes only accounted for 4.2% of the assembly process of specific taxa from water to gut in CK, but this was increased to 72.2% and 91.7% in CN10 and CN15, respectively (Supplementary Fig. 11d). Niche breadths were significantly different among total, core and specific OTUs, being highest for core OTUs and lowest for specific OTUs (Supplementary Fig. 12).

## Discussion

Several studies have revealed that shrimp gut microbiota is closely associated with the environmental microbiota in shrimp culture systems, especially in biofloc-based culture systems^9,10^. However, little is known about the extent to which the environmental microbiota influences changes in the intestinal microbiota in different culture systems. In this study, we analyzed the relationships among bacterial communities in water, biofloc, and gut in control and sucrose addition groups. We found that bacterial communities in water, biofloc, and gut in the control group were significantly separated, while they were gathered together in sucrose addition groups. Sucrose addition markedly increased the aggregation of core taxa, which was closely linked to bacterial network stability of the shrimp culture system and directional recruitment of specific taxa for shrimp growth. Below, we discuss how these results have widened our knowledge of sucrose addition-induced changes in composition, stability, and ecological processes of bacterial communities in shrimp culture systems.

### Sucrose addition increases bacterial community similarity in shrimp culture systems

It has been widely reported that the addition of carbon sources, such as glucose^23^, starch^24^, and cassava dregs^25^, significantly changes the bacterial community structure and composition of shrimp culture systems. Our recent study found that the bacterial community in rearing water was rapidly changed once glucose was added, while that in the gut was gradually influenced over time^23^. In the present study, we found that sucrose addition not only affected bacterial community structure in water, biofloc, and gut but also influenced the relationships among them. The dissimilarities among bacterial communities in water were significantly increased by sucrose addition, while they were markedly reduced among gut communities, demonstrating that sucrose addition enhances variations in bacterial communities in water but stabilizes community structure in the gut (Fig. 1). The microbial community in water is closely associated with environmental factors, and the addition of a carbon source into water may randomly increase the enrichment of certain bacteria in each tank^8,23^, thus boosting variations in the bacterial communities within groups after sucrose addition.

Meanwhile, the relationships among bacterial communities in the gut, biofloc, and rearing water were different in the control culture system and that with continuous sucrose addition. Pairwise dissimilarities in the bacterial community in gut, biofloc, and rearing water were significantly decreased by sucrose addition (Fig. 1b), further indicating that sucrose addition has a consistent influence on bacterial communities in the shrimp culture system. Similarly, inputs of organic matter and other human interventions can directionally affect bacterial community structure and composition of shrimp culture systems^10^. The bacterial communities in biofloc and gut were more similar than those in water and gut (Table 1 and Fig. 1), indicating that the bacterial community in biofloc has a more important role in shaping the gut microbiota. This phenomenon was also found in a natural shrimp culture system where the bacterial community of shrimp gut was more similar to that in sediment rather than water^10,26^. Our previous study also found that bacteria from bioflocs, especially large-size bioflocs, are closely associated with the shrimp gut microbiota in biofloc-based culture systems^9^. Bioflocs are an ideal natural food for shrimp ingestion^27^, so shrimp excretion and various microbes in bioflocs might increase the interaction of bacterial communities in the gut and bioflocs, making the bacterial community of the gut more closely associated with that of bioflocs than that of water. In addition, previous studies found that a combination of commercial feed with biofilms shapes the gut microbiota of shrimp^28^. Bioflocs in the shrimp culture system include a lot of biofilm-producing bacteria, which might play an important role in shaping the gut microbiota.

The microbial community in shrimp gut is not the same as those in surrounding water in clear seawater culture systems, and carbon source addition can synchronously modify the microbial communities of water and gut in biofloc-based culture systems^8,26^. The numbers of OTUs shared by the three habitats (water, bioflocs, and gut) were 1.6–1.9 times higher in sucrose addition groups than in the control group (Fig. 2a), revealing that the increased similarity in sucrose addition groups was closely associated with bacterial community composition. The relative abundances of dominant shared taxa in water, bioflocs and gut were obviously different in the control culture systems, especially in gut, which possessed more opportunistic pathogens such as *Vibrio*, *Photobacterium,* and *Candidatus* Bacilloplasma. However, in sucrose addition groups, the relative abundances of dominant shared taxa became more consistent (Fig. 2b), and mainly consisted of Actinobacteria (mostly belonging to Microbacteriaceae and Demequinaceae), Rhodobacteriaceae and Flavobacteriaceae in the three habitats, further indicating that the interactions of the bacterial community in the three habitats were enhanced by sucrose addition. SourceTracker analysis implied that bioflocs were important for mediating interactions between water and gut bacterial communities. Although more than 90% of the bacteria in the bioflocs were sourced from water in both control and sucrose addition culture systems, the contributions of water and bioflocs to the shrimp gut bacterial community were significantly enhanced by sucrose addition. The small-sized bioflocs and low biomass of bioflocs in the control culture system may limit the interactions between bacterial communities in the surrounding environment and gut^7,9^, thus reducing the transfer of bacteria from water to gut. This is in agreement with observations that shrimp source little of their gut microbiota from their rearing water in culture systems without bioflocs^26^, and that gut bacterial communities are more similar to those in the surrounding environment in culture systems with high biofloc biomass^7^.

To understand which taxa were specifically affected by sucrose addition, we divided the bacterial community into core taxa and specific taxa. Interestingly, sucrose addition had a strong effect on the composition and abundance of core taxa and specific taxa in water and biofloc, but mainly changed the composition and abundance of core taxa in the gut (Figs. 2d and 3). Specific taxa were largely replaced by core taxa after sucrose addition in both water and biofloc samples, and these enriched core taxa were further enriched in the gut (Fig. 3c), thus making the compositions of bacterial communities more similar in the three habitats.

### Core taxa are more important for maintaining bacterial network stability of shrimp culture systems and shrimp growth

The ecological stability of the culture system is considered critical for host health because it ensures that beneficial symbionts and their related functions are maintained over time^29,30^. Microbial communities in ecosystems are key drivers of many ecosystem processes and play important roles in sustaining ecological stability^29^. Microbial co-occurrence networks provide comprehensive insight into microbial community structure and assembly patterns, and network properties such as modularity and cohesion values have been used to evaluate microbial community stability in soil^31,32^, plants^33^ and marine ecosystems^34^.

This study demonstrated that sucrose addition increases the bacterial community stability of a shrimp culture system according to the following three factors. First, modularity reflects interactive relationships spatial compartmentalization, and yielding non-random patterns of network structure, and thus largely affects the stability of microbial communities^31^. High modularity limits the influence on its own module of losing a taxon and reduces the effect on the rest of the network, thus helping to maintain a stable community^29^. In this study, sucrose addition increased the modularity of the bacterial community network of the shrimp culture system, indicating that the stability of the microbial community might be improved by sucrose addition. Second, cooperation leads to coupling between species and positive feedback, thus destabilizing the microbial community; meanwhile, competition promotes stability by restraining these positive-feedback loops^28^. Weakened interactions between species will generally promote the stability of a microbial community by reducing the coupling caused by strong cooperative interactions^29^. Therefore, the strength and number of positive and negative associations are closely associated with microbial stability^29^. Here, the cohesion value was used to estimate the influence of sucrose addition on the bacterial stability of the culture system. Previous studies indicated that the cohesion value represents the strength and ratio of positive and negative associations in the microbial network^35^ and has been successfully applied in evaluating the effects of environmental stress and climate warming on microbial network stability^31,32^. Sucrose addition significantly increased negative:positive cohesion by largely reducing the positive cohesion (Fig. 4a), demonstrating that cooperative interactions might be limited or weakened by sucrose addition, thus enhancing the bacterial network stability of the culture system. Third, natural connectivity has been proposed for sensitively assessing the robustness of complex networks^36^. Higher natural connectivity reveals that more alternative routes are available for maintaining communication between taxa when a network is destroyed, thus maintaining the stability of the microbial network^36^. Here, we found that the natural connectivity was always higher in the sucrose addition groups than in the control group (Supplementary Fig. 6c), again confirming that sucrose addition stabilizes the bacterial community of the shrimp culture system.

Previous studies showed that core taxa were more stable in a microbial community^37,38^. We found that core taxa shared by the three culture systems possessed higher negative:positive cohesion and natural connectivity in the sucrose addition groups (Fig. 4a and b), indicating that the stability of core taxa was largely increased by sucrose addition. Meanwhile, the core taxa had a higher niche breadth than total or specific taxa (Supplementary Fig. 12), demonstrating that core taxa can adapt to a wide range of environmental niches, thus contributing to maintaining the equilibrium of microbial communities after perturbation^38,39^.

Microbial keystone taxa are highly connected taxa that have a key role in microbial communities, and their removal can result in enormous variations in microbiome structure and functioning^18^. Our data showed that the sucrose addition networks not only possessed more keystone taxa than the control network, but also showed significantly higher relative abundances of certain keystone taxa (Fig. 5 and Supplementary Fig. 7), highlighting the importance of these taxa for network stability. It should be noted that although the compositions were different, most keystone OTUs were identified as core taxa in both control and sucrose addition networks, suggesting that the core keystone taxa are crucial for microbiome functioning. It has been reported that some of the keystone taxa are part of the core microbiome in plant roots^40^, and the contribution of keystone taxa in stabilizing a microbial community will be higher if they are part of the core microbiome and consistently present in an environment^18,41^. Therefore, more core keystone taxa in the sucrose addition networks may also make their communities more stable.

The majority of these keystone OTUs in the sucrose addition networks were assigned to three phyla of Actinobacteria, Alphaproteobacteria (mostly belonging to Rhodobacteraceae), and Planctomycetes (mainly belong to the genera *Blastopirellula*, *Planctomyces,* and *Rhodopirellula*). Members of the Actinobacteria are generally producers of antibacterial and growth-promoting substances^42^, and Rhodobacteraceae are generally considered to be a potential probiotic group in the shrimp culture system due to their various functions in the removal of nitrogen and the production of vitamins and antibacterial substances^43^. Therefore, the keystone OTUs in these phyla may be closely associated with the healthy growth of shrimp. Our data showed that the relative abundances of two Actinobacteria OTUs (OTU2327 and 6326) and one Rhodobacteraceae OTU1867 were significantly increased by sucrose addition, and showed significant and positive associations with SGR, SR, and yield of shrimp (Fig. 5d), highlighting their importance in promoting shrimp health. It should be noted that the keystone taxa in sucrose addition networks tended to recruit taxa belonging to Actinobacteria no matter which phylum the keystone taxa belonged to (Fig. 5c). Moreover, these keystone taxa not only recruited the core Actinobacteria taxa but also specific Actinobacteria taxa. One example of this is that the keystone OTU6326 recruited three core Actinobacteria taxa and five specific Actinobacteria taxa, which also showed significant and positive associations with shrimp growth parameters, especially in the gut (Supplementary Fig. 8). This is consistent with keystone taxa or members of keystone guilds potentially being functionally redundant^18^. It has to be noted that the bacterial community of control group might be affected by the massive mortality of shrimps and extensive water exchange at 11–13th day, as well as the deserted biofloc and water samples due to the poor DNA quality. We are not sure that to what extent these changes affect the bacterial community of control group and the overall comparative analysis with sucrose addition groups. These changes are most likely to impact the correlations between bacterial taxa and shrimp growth parameters, so further study is necessary to understand the causality, in terms of the influence of keystone taxa and their accessories on microbiome functioning and host health.

### Deterministic selection dominates bacterial community assembly from water and biolfoc to gut after sucrose addition

A number of studies have reported that adding exogenous additives (e.g., carbon sources, probiotics, etc.) into the culture system can reshape the gut microbiota of aquatic animals through manipulating the water microbial community^6,44^. However, how these exogenous additives affect the assembly process of microbial communities from environment to gut is still poorly understood. In this study, sucrose addition made the assembly of the bacterial community from the environment to gut more deterministic. This might explain why the bacterial communities with sucrose addition were more similar between water or biofloc and gut than those without sucrose, and also confirms that more bacterial communities in the gut are sourced from water and biofloc in the sucrose addition groups than in the control group. The deterministic transfer of bacteria from environment to host in sucrose addition groups may be highly associated with the aggregation of *K-strategist* bacteria. It has been reported that microbial communities in biofloc-based culture systems are dominated by *K-strategists*^5^, which establish themselves more slowly and have a higher chance of coevolving^45^. Members of Actinobacteria and Rhodobacteraceae are predominantly *K-strategists*^6,46^, and their relative abundances of them were also simultaneously increased in water, biofloc, and gut (Fig. 3c), highlighting the importance of deterministic assembly of bacterial communities from the surrounding environment to the gut. Complex nutrients including residual feed, shrimp feces, exuviate, dead microbes and additional carbon sources can cause a high solids concentration, which might lead to strong selection pressure for restraining fast-growing (*r-strategists*) microbes in the sucrose addition groups^5,6^. By contrast, the control culture system may facilitate the growth of *r-strategist* bacteria without strong selection pressure for any particular taxa, thus resulting in a more random assemblage^45^.

It should be noted that the assembly processes of core taxa from the environment to gut were dominated by deterministic selection in both control and sucrose addition groups, but sucrose addition significantly increased the selection process of specific taxa from environment to gut, especially from water to gut (Supplementary Fig. 11). The host favors recruitment of key microbial taxa to ensure key functions for holobiont fitness and health of the components of the holobiont, and the core microbiome generally includes many key microbial taxa, which might be directionally recruited from the environment^18,47^ and thus mainly dominated by the deterministic assembly process. The core taxa can positively recruit certain specific taxa for host growth, such as OTU6326 in this study, and sucrose addition increases positive associations between core taxa and specific taxa (Supplementary Table 3). These results might explain the boosted deterministic transfer of specific taxa from the environment to host, although further investigation is needed.

In summary, this study systematically evaluated the effects of sucrose addition on the interaction, network stability, and assembly mechanisms of bacterial communities in a shrimp culture system. The results indicated that sucrose addition largely increases the migration of bacterial communities from water and biofloc to gut, thus making the bacterial communities in the culture system more similar. Core taxa were consistently enriched in the water, biofloc, and gut by sucrose addition and played crucial roles in stabilizing the bacterial network of the culture system. The majority of keystone taxa belonged to the core taxa, and three keystone OTUs were either directly associated with shrimp growth or indirectly linked to shrimp health by recruiting certain specific taxa. Moreover, the ecological migration of bacterial communities from water and biofloc to gut was mainly governed by heterogeneous selection in the sucrose addition systems. These results are helpful for establishing microbial ecological strategies for directionally regulating bacterial communities for healthy shrimp growth.

## Methods

### Experimental design and sample collection

The experimental procedures were approved by the Local Institutional Animal Care and Use Committee (approval number: 10258), and were performed in the Yongxing Base of Zhejiang Mariculture Research Institute, Zhejiang, China. Healthy juvenile shrimps with similar weight (~4.3 g) were obtained from Zhejiang Mariculture Research Institute and were housed in 18 conical tanks filled with 400 L sanitized seawater with a density of 210 shrimps per tank. After adaptation to experimental conditions for two days, these tanks were randomly divided into three groups (six replicates per group): control group, feeding basal diet (C/N ratio of ~6:1, 44.20%, w/w, carbon and 7.04%, w/w, nitrogen; AlphaFeedCo., Ltd., Shenzhen, China); CN10 group, feeding the mixture of basal diet and sucrose (42.1%, w/w, carbon, purity 99%) with a ratio of 5:3 to obtain a C/N ratio of ~10:1; CN15 group, feeding the mixture of basal diet and sucrose with a ratio of 2:3 to obtain a C/N ratio of ~15:1. Previous studies indicated that a C/N ratio in the substrate ranging from 10 to 20 is considered optimal for heterotrophic bacteria to assimilate NH_4_-N^48–50^. When the C/N ratio is between 8 and 10, it depends on the joint action of autotrophic microorganisms and heterotrophic microorganisms to remove inorganic nitrogen; but when the C/N ratio reaches 15, inorganic nitrogen can basically be consumed by the assimilation of heterotrophic microorganisms^51,52^. Therefore, we selected two ratios (CN10 and CN15) to study their impact on bacterial communities of culture systems.

The feeding trial lasted for 28 days with three times per day (07:00 am, 12:00 pm, and 17:00 pm). The water was not exchanged for the first week in all experiment tanks, and then an average 5% and 2% water exchange was performed every day after one week in control (CK) and sucrose addition (CN10 and CN15) groups, respectively. Due to the massive death of shrimps in two tanks from the CK group on day 11, an emergency measure with 20% water exchange was performed on days 11 and 12, and 10% on day 13 in CK group. Bioflocs were universally formed by stimulating the growth of heterotrophic microbiota in CN10 and CN15 groups after 10 days of cultivation. Constant aeration was provided in all experimental tanks using air stones during the entire experiment, and the water lost due to natural evaporation was compensated daily using sterile distilled water after water exchange. Except for the above differences, basic management practices were the same for all experimental groups (water temperature: 26 ± 3 °C; pH: 7.7 ± 0.3; Dissolved oxygen: 6.5 ± 0.5 mg L^−1^). At the end of the experiment, the rearing water from each tank was first filtered through a 100 μm filter to collect biofloc samples. Then, one part of the rearing water was filtered again through a 0.45 μm polycarbonate membrane to measure water quality parameters. Another part was filtered through a 0.22 μm polycarbonate membrane, and trapped solids were regarded as water samples. Five shrimps from each tank were randomly selected for sampling, and their guts were dissected using sterile instruments. All above samples were frozen quickly and stored at −80 °C for bacterial community analysis. Sampling was performed after a 15 h fasting period.

### Assessment of water quality, biofloc contents, and shrimp growth performance

Water quality parameters comprising ammonium-nitrogen (NH_4_-N), nitrite-nitrogen (NO_2_-N), nitrate-nitrogen (NO_3_-N), phosphate-phosphorus (PO_4_-P), chemical oxygen demand (COD) and chlorophyll a (Chl-a) were measured according to standard methods (GB17378.4-2007). Biofloc volume was determined using an Imhoff cone (1000-0010, Nalgene) at the end of the experiment according to our previous description^19^. Growth performance such as survival rate (SR), specific growth rate (SGR) and total yield were acquired by calculating numbers and weight of shrimps at the end of the experiment.

### DNA extraction, 16S rRNA gene sequencing, and data processing

Bacterial DNA was isolated from water and biofloc samples using a Power Soil® DNA isolation kit (MOBIO, USA) and from gut samples using a QIAamp® DNA Stool Mini Kit (Qiagen, Germany) following the manufacturer’s instructions, respectively. The concentrations and quality of DNA were measured using a Nanodrop 2000 spectrophotometer (NanoDrop Technologies, DE, USA), and DNA was stored at −20 °C for further analysis. Since the DNA concentrations of two water samples and four biofloc samples in control groups were unqualified, a total of 48 DNA samples, including 16 from water, 14 from bioflocs, and 18 from gut samples, were used in subsequent experiments. The V4 region of the 16S rRNA gene was amplified using primers 515F-Y (5′-GTGYCAGCMGCCGCGGTAA-3′) and 806R (5′-GGACTC ANVGGGTWTCTAAT-3′). PCR products were purified using a PCR fragment purification kit (TaKaRa, Japan), then quantified using an Agilent 2100 Bioanalyzer (Agilent, USA) and sequenced on the Illumina MiSeq platform (Illumina, USA).

Raw data were processed and analyzed using Quantitative Insights Into Microbial Ecology (QIIME 1.9.1, http://qiime.org/)^53^. Quality control and chimaera removal of sequences were performed using USEARCH^54^. The remaining sequences were clustered into operational taxonomic units (OTUs) at 97% sequence similarity cutoff using the *pick_open_reference_otus.py* script. A representative sequence for each OTU was annotated taxonomically based on the SILVA 128 database, and all singletons and OTUs associated with chloroplasts or mitochondria and all unassigned and unclassified sequences were removed from the data. To correct unequal sequencing depth, the OTU table was rarefied at 18,400 sequences per sample for downstream analysis.

### Diversity and taxonomic analysis

α-Diversity indexes (richness, evenness, and phylogenetic diversity) were calculated using QIIME 1.9.1. The variable coefficient was calculated by dividing the standard deviation by mean value of each α-diversity indexes within group. Principal coordinate analysis (PCoA), analysis of similarity (ANOSIM), and permutational multivariate analysis of variance (PERMANOVA) were carried out to evaluate the overall differences in bacterial communities among groups based on the Bray-Curtis dissimilarity. Shared OTUs among different groups were determined using Venn diagram analysis with the online jvenn tool (http://jvenn.toulouse.inra.fr/app/example.html). SourceTracker analysis was applied to estimate the effects of sucrose addition on the sources of bacteria in biofloc and gut samples, as described by Knights et al.^55^. OTUs were classified as core and specific OTUs to understand the effects of sucrose addition on the bacterial communities of the culture systems. The 258 core OTUs were defined as OTUs occurring simultaneously in water, biofloc, and gut samples of CK, CN10, and CN15 groups. The specific OTUs were divided into group-specific OTUs, defined as OTUs shared by water, biofloc, and gut samples in any one or two groups, and sample-specific OTUs occurring only in water, biofloc, or gut samples or two of these regardless of group. Differentially abundant OTUs were identified at false discovery rate (FDR) corrected *p* < 0.05 between the sucrose addition group and the control group using the “DESeq2” package in R 4.0.3^56^.

### Bacterial community network construction and characterization

To investigate how the bacterial community structures of the shrimp culture system changed after sucrose addition, co-occurrence networks for CK, CN10, and CN15 were constructed using SparCC^57^ with average reads ≥5 for each OTU. SparCC analysis was performed to calculate robust correlations with a median of 20 iterations, and pseudo *p*-values with 100 bootstrap samples were inferred using the “SpiecEasi” package in R 4.0.3. Robust correlations (|ρ| ≥ 0.6, *p* < 0.05) between OTUs were selected and visualized using Gephi 0.9.2^58^. The stability of each network was evaluated by calculating robustness and cohesion values. Robustness was used to estimate network stability by removing nodes in the static network to assess how quickly the natural connectivity decreased^59^. The natural connectivity is expressed in mathematical form as a special case of average eigenvalue and it characterizes the redundancy of alternative paths by quantifying the weighted number of closed walks of all lengths. This measure is defined as an “average eigenvalue” of the graph adjacency matrix^59^:

1 $$\bar \lambda = {{{\mathrm{ln}}}}\left( {\frac{1}{n}\mathop {\sum}\limits_{i = 1}^N {e^{\lambda i}} } \right)$$

where *N* is the number of nodes in a network G and λi is the ith element of the set {λ1, λ2,…, λN}, which is called the spectrum of G. Cohesion is a recently developed metric that can quantify the degree of community complexity^30,34^. Two cohesion values (positive and negative) were calculated for each sample as the sum of the significant positive or negative correlations between taxa multiplied by taxa abundances according to the method of Herren and McMahon^35^:

2 $${{cohesion}} = \mathop {\sum}\limits_{{\it{i}} - 1}^{\it{m}} {{{abundance}}_{\it{i}} \times {{connectedness}}_{\it{i}}}$$

where *m* is the total number of taxa in a community. The degree of bacterial network stability is strongly associated with the proportion of negative to positive co-occurrences as the absolute value of negative:positive cohesion. A higher absolute value of negative:positive cohesion represents more stability of the bacterial network^30,31^.

To identify the keystone OTUs of each network, the connectivity of each node was assayed based on its within-module connectivity (Zi) and among-module connectivity (Pi)^60^. Keystone OTUs were classified as three types: module hubs (highly connected nodes within modules, Zi > 2.5), network hubs (highly connected nodes within the entire network, Zi > 2.5 and Pi > 0.62), and connectors (nodes that connect modules, Pi > 0.62)^60^. Correlations between the keystone OTUs of each network or OTUs they recruited and water quality parameters, biofloc content, and shrimp growth performance indexes were calculated using pheatmap based on Pearson’s correlation analysis. Recruitment networks for keystone OTUs were constructed using Cytoscape 3.3.0^61^.

### Estimation of ecological processes

To reveal the assembly process of bacterial communities from water to biofloc and gut, and from biofloc to gut, the β-nearest taxon index (βNTI) and Bray–Curtis-based Raup–Crick index (RC_bray_) were calculated using the “picante” package in R 4.0.3^62^. Ecological processes were divided into deterministic (heterogeneous select and homogeneous select) and stochastic (dispersal limitation and homogenizing dispersal) process according to βΝΤΙ and RC_bray_ values^22^. The neutral community model was used to test the effects of neutral processes on bacterial community assembly according to the description of Sloan et al.^63^. The 95% confidence interval was estimated to classify OTUs as neutrally distributed or above or below predictions according to the frequency of OTU occurrence and their relative abundance using the water or biofloc bacterial community as a pool. The *R*^2^ value showed the goodness of neutral model fitting. When *R*^2^ is >0, community assembly is consistent with a neutral process, but when it is ≤0, community assembly cannot be described by a neutral process. The estimated migration rate (*m*) is the measure of dispersal limitation, and higher m values indicate less dispersal limitation^64^. The Levins’ niche breadth (B) index of each OTU was calculated using the “spaa” package in R 4.0.3^65^.

### Statistical analysis

Statistical analyses were performed by using the “VEGAN” package in R 4.0.3 to assess the significant differences between groups, including water quality parameters, growth parameters, α-diversity indexes, the distance between samples, bacterial abundance, SourceTracker analysis, cohesion values and β-NTI values based on Student’s *t* test.

### Reporting summary

Further information on research design is available in the Nature Research Reporting Summary linked to this article.

## Supplementary information

Supporting information

Reporting Summary

Supporting information

Supporting information

## Data Availability

All sequencing data generated in this study are publicly available from the Genome Sequence Archive in the BIG Data Center, Chinese Academy of Sciences (http://bigd.big.ac.cn/gsa, accession number: CRA005186 and CRA005187).
