# Supplementary material for: Sucrose addition directionally enhances bacterial community convergence and network stability of the shrimp culture system
Source: NPJ Biofilms Microbiomes. 2022 Apr 11;8:22. doi: 10.1038/s41522-022-00288-x (PMC9001642; doi:10.1038/s41522-022-00288-x)
Supplement: Supplementary file 1 — Supporting information [file 41522_2022_288_MOESM1_ESM.pdf]

Sucrose addition directionally enhances bacterial community convergence and network stability of the shrimp culture system

Haipeng Guo[1,2*], Pengsheng Dong[1,2], Fan Gao[1,2], Lei Huang[1,2], Sipeng Wang[1,2], Ruoyu Wang[1,2], Mengchen Yan[1,2], Demin Zhang[1,2*]

[1] State key laboratory for managing biotic and chemical threats to the quality and safety of agro-products, Ningbo University, Ningbo 315211, China

[2] School of Marine Sciences, Ningbo University, Ningbo 315211, China

Supplementary Table 2 Topological properties of networks associated with CK, CN10 and CN15 culture systems.

Group	Numbers of nodes	Numbers of edges	Average connectivity	Graph density	Modularity	Average path length	Average clustering coefficient
CK	231	1327	5.744	0.072	1.665	3.099	0.539
CN10	249	1100	4.418	0.046	1.752	3.642	0.428
CN15	247	1246	5.045	0.055	1.858	3.257	0.507

Supplementary Table 3 The positive and negative associations between core and core, core and specific, and specific and specific taxa in the CK, CN10 and CN15 bacterial networks.

Taxa	Edges	CK		CN10		CN15	
		Positive (Ratio, %)	Negative (Ratio, %)	Positive (Ratio, %)	Negative (Ratio, %)	Positive (Ratio, %)	Negative (Ratio, %)
/	Total	849 (64.0)	477 (36.0)	724 (66.0)	376 (34.0)	790 (63.4)	356 (36.6)
Total to total	Core to core	427 (32.2)	92 (7.0)	315 (28.6)	83 (7.5)	274 (22.0)	101 (8.1)
	Core to specific	253 (19.1)	308 (23.2)	234 (21.3)	210 (19.1)	267 (21.4)	292 (23.4)
	Specific to specific	169 (12.7)	77 (5.8)	175 (15.9)	83 (7.5)	249 (20.0)	63 (5.0)
Keystone to total	Core to core	74 (5.6)	8 (0.6)	66 (6.0)	3 (0.3)	94 (7.5)	16 (1.3)
	Core to specific	11 (0.83)	16 (1.21)	6 (0.55)	17 (1.55)	18 (1.44)	47 (3.77)
	Specific to specific	4 (0.30)	1 (0.08)	2 (0.18)	2 (0.18)	29 (2.33)	32 (2.57)
Keystone to keystone	Core to core	4 (0.30)	0	3 (0.27)	0	10 (0.80)	0
	Core to specific	0	0	0	2 (0.18)	0	6 (0.48)
	Specific to specific	0	0	0	0	1 (0.08)	0

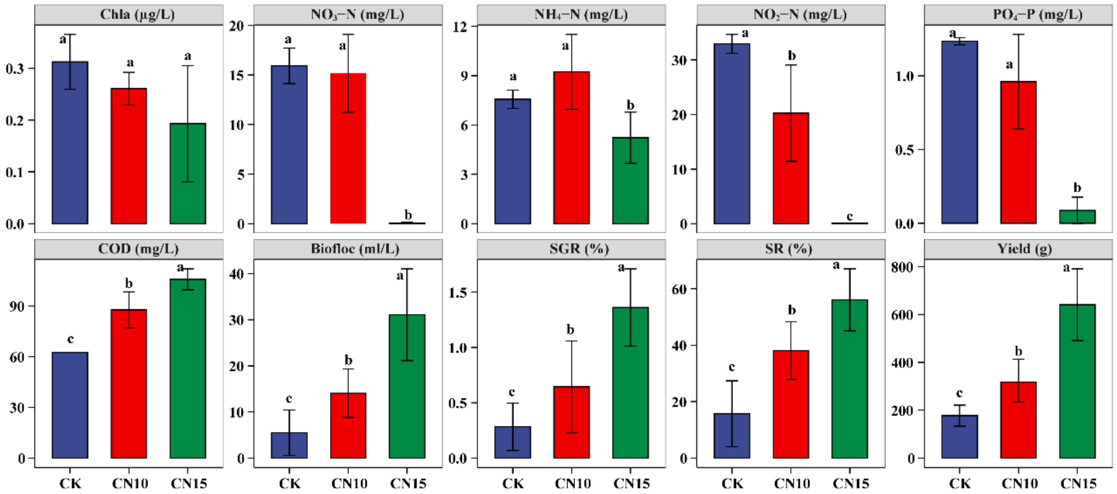

Supplementary Fig. 1 Effects of sucrose addition on the water quality parameters, biofloc volume, and shrimp growth performances. NH_4^+-N, ammonium-N; NO_2^--N, nitrite-N; NO_3^--N, nitrate- N; PO_4^--P, phosphate-P; COD, chemical oxygen demand; Chl-a, Chlorophyll a; SR, survival rate; SGR, specific growth rate; Yield, total yield of shrimp each pond. Each sample had six replicates (n=6), and error bars show standard deviation. Different letters indicate a significant difference at $p < 0.05$ based on Student's t test. CK: without sucrose addition; CN10: the C/N ratio of about 10:1 obtained by sucrose addition; CN15: the C/N ratio of about 15:1 obtained by sucrose addition.

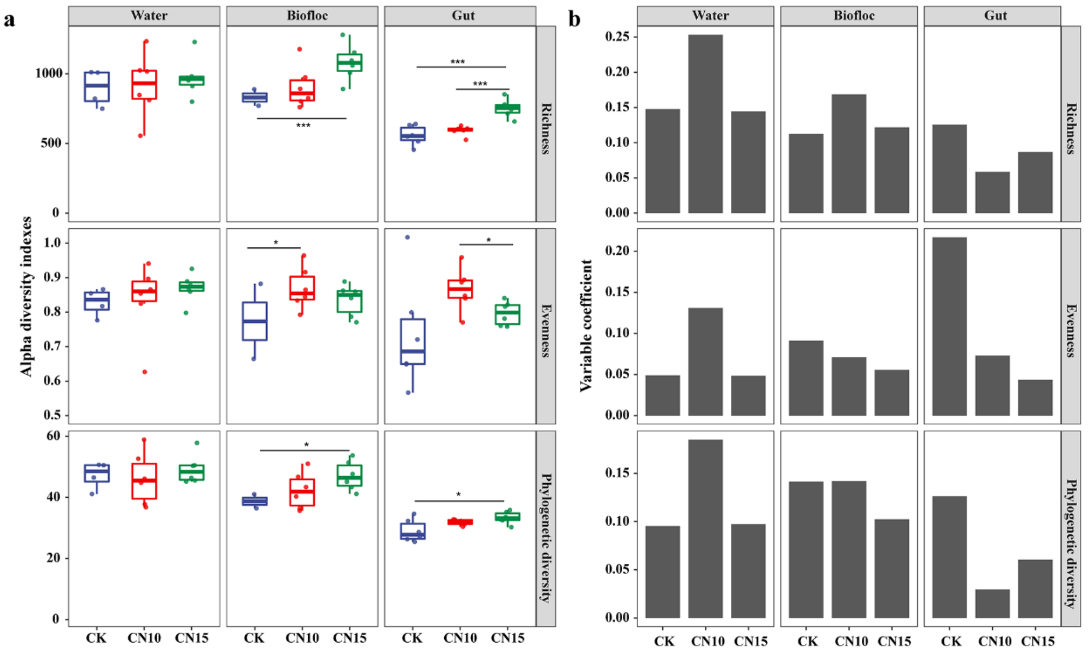

Supplementary Fig. 2 Effects of sucrose addition on the α-diversity characteristics of the bacterial communities in rearing water, bioflocs, and gut. a Effects of sucrose addition on α-diversity indexes, including richness, evenness and phylogenetic diversity. The boxes represent the median and interquartile range, and whiskers range from minimum to maximum values. Different asterisks indicate a significant difference at *$p < 0.05$, **$p < 0.01$, and ***$p < 0.001$ based on Student's t test. **b** Effects of sucrose addition on the variable coefficient of α-diversity indexes. CK: without sucrose addition; CN10: the C/N ratio of about 10:1 obtained by sucrose addition; CN15: the C/N ratio of about 15:1 obtained by sucrose addition. Each sample had six replicates (n=6), except the water samples (n=4) and biofloc samples (n=2) in control group.

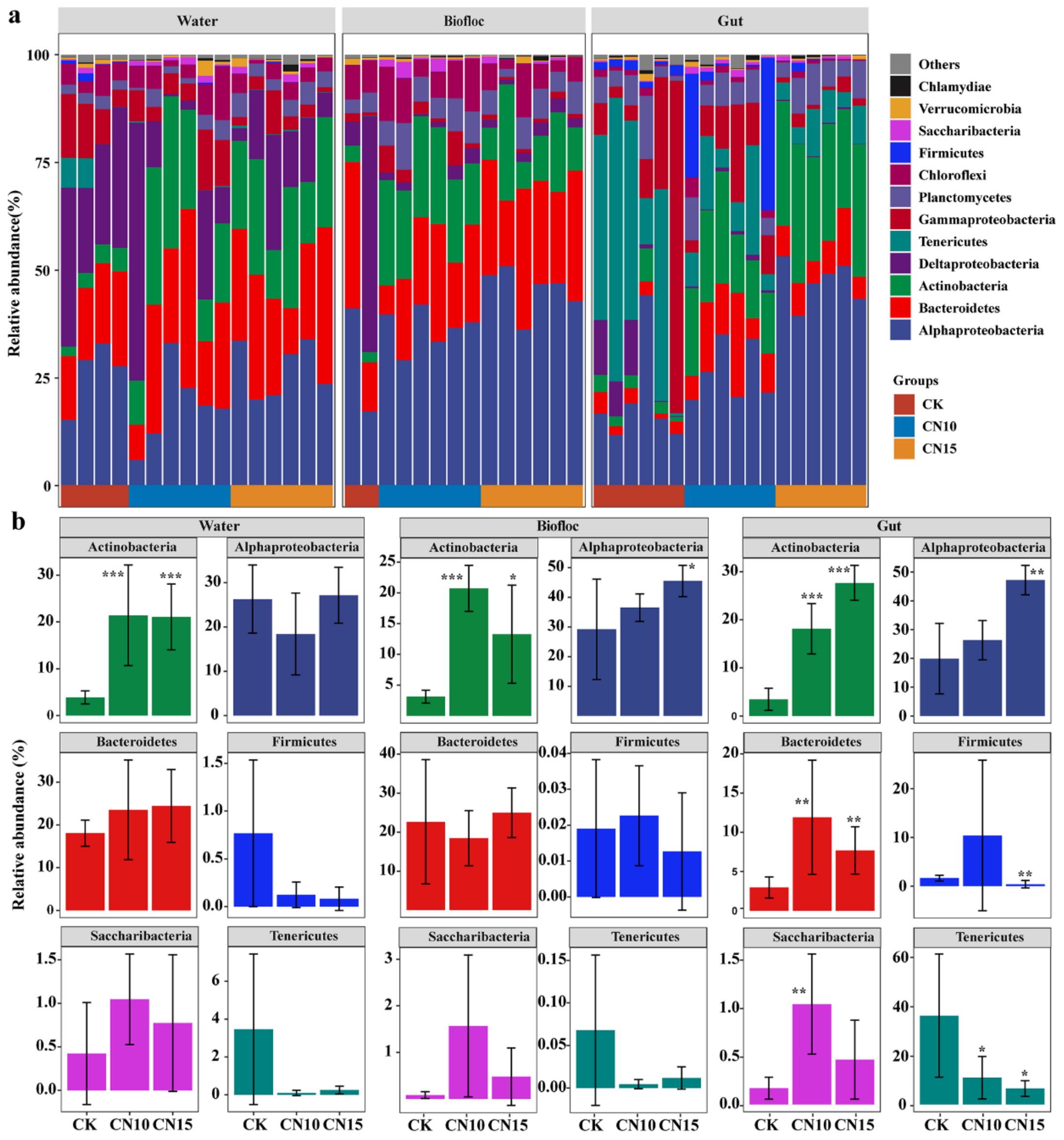

Supplementary Fig. 3 Relative abundance of the dominant phyla/Proteobacteria classes between control and sucrose addition groups. a Relative abundance in rearing water, bioflocs and gut. **b** Key phyla/Proteobacteria classes showing significant differences. Error bars show standard error. Different asterisks indicate a significant difference at *$p < 0.05$, **$p < 0.01$, and ***$p < 0.001$ based on Student's t test. CK: without sucrose addition; CN10: the C/N ratio of about 10:1 obtained by sucrose addition; CN15: the C/N ratio of about 15:1 obtained by sucrose addition. Each sample had six replicates (n=6), except the water samples (n=4) and biofloc samples (n=2) in control group.

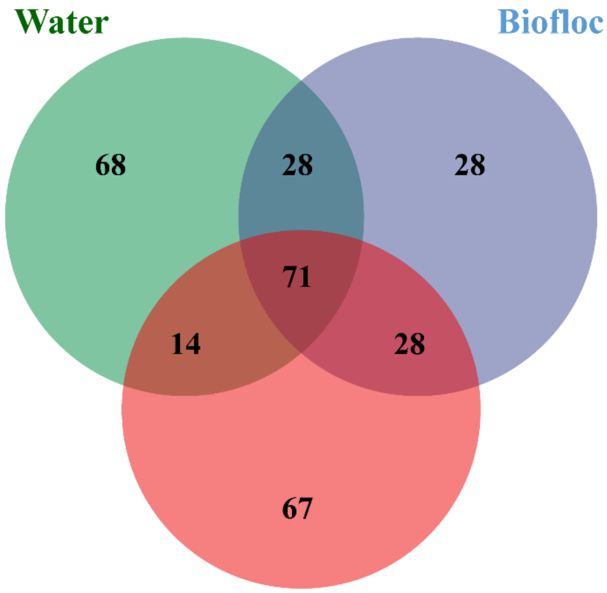

Supplementary Fig. 4 Venn analysis of the numbers of significantly discriminatory OTUs (P_{FDR} < *0.05*) shared by water, bioflocs and gut between control and sucrose addition groups.

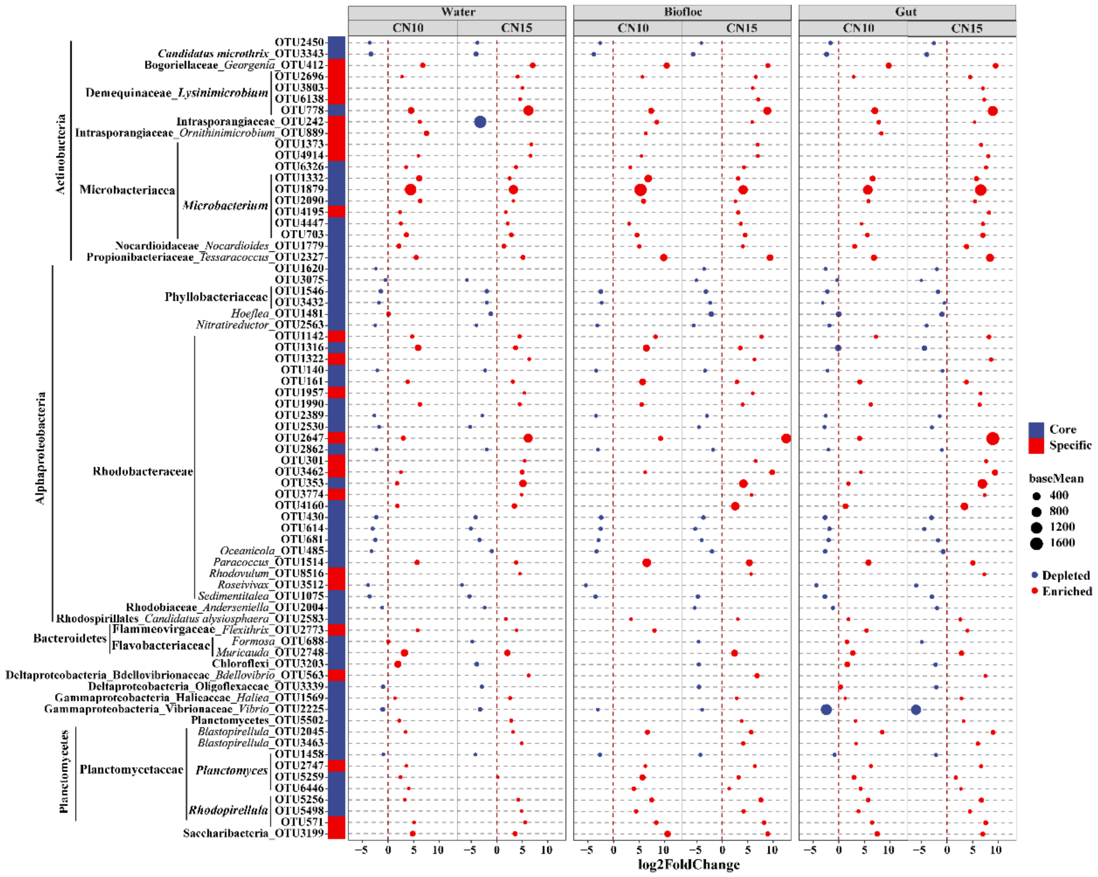

Supplementary Fig. 5 DESeq2 showing the significantly enriched and depleted OTUs (P_{FDR} < *0.05*) shared by water, bioflocs and gut in sucrose addition groups, compared to that in control group. The size of the point represents the average normalized abundance. The blue square indicates that the discriminatory OTUs are assigned as core taxa, and the red square as specific taxa. CK: without sucrose addition; CN10: the C/N ratio of about 10:1 obtained by sucrose addition; CN15: the C/N ratio of about 15:1 obtained by sucrose addition. Each sample had six replicates (n=6), except the water samples (n=4) and biofloc samples (n=2) in control group.

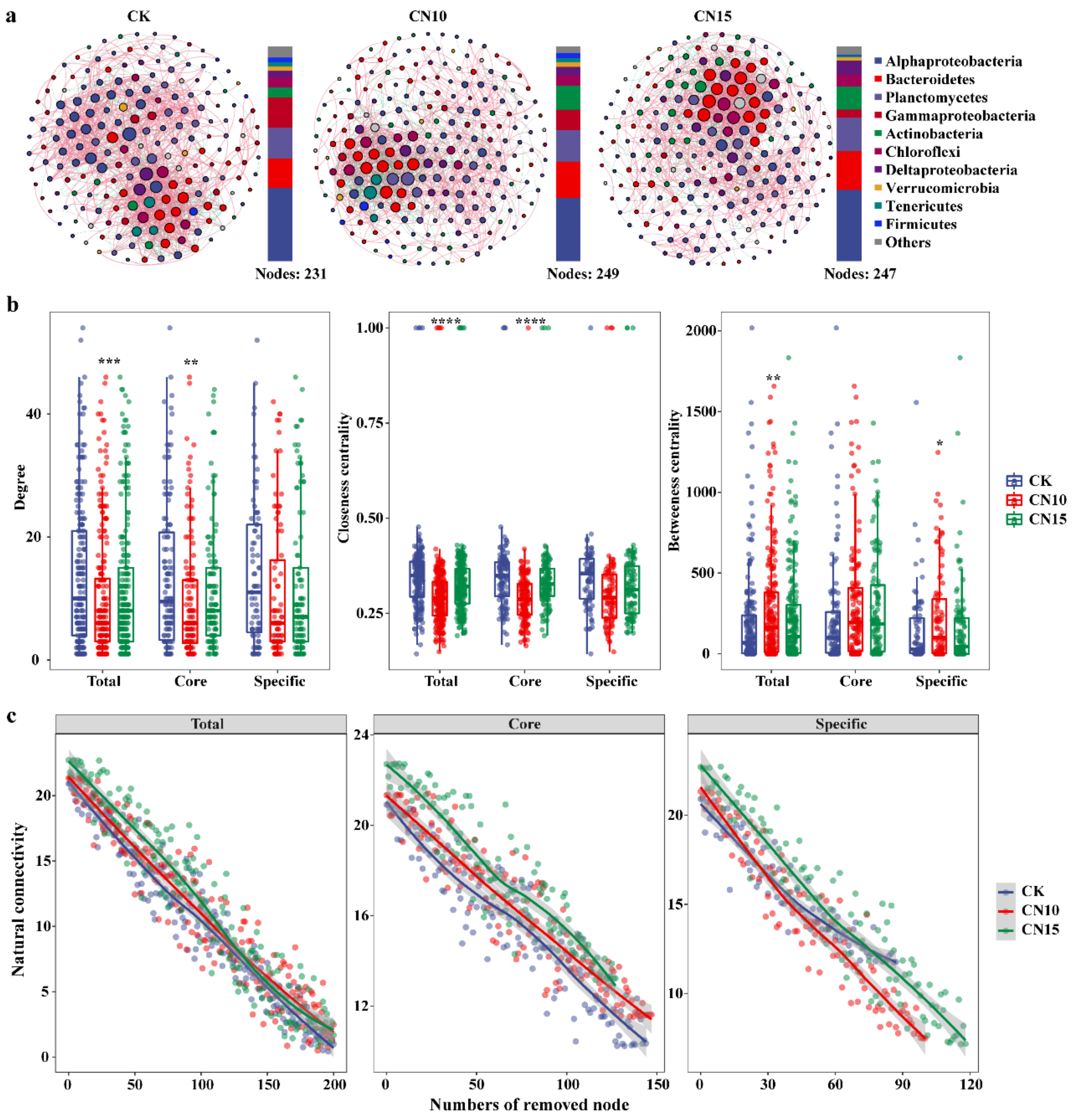

Supplementary Fig. 6 Co-occurrence networks and their characteristics in control and sucrose addition culture systems. a Co-occurrence networks of bacterial OTUs and their node composition in CK, CN10 and CN15 groups. A connection represents a strong (Robust correlations |ρ|≥0.6) and a significant (infer pseudo p values < 0.05) correlation. The size of each node is proportional to the number of degrees. **b** The degree, closeness centrality and betweeness centrality of total, core and specific taxa. The boxes represent the median and interquartile range, and whiskers range from minimum to maximum values. Different asterisks indicate a significant difference at $*p < 0.05$, $**p < 0.01$, and $***p < 0.001$ based on Student's t test. **c** The robustness (natural connectivity) of total, core and specific taxa in control and sucrose addition groups. CK: without sucrose addition; CN10: the C/N ratio of about 10:1 obtained by

sucrose addition; CN15: the C/N ratio of about 15:1 obtained by sucrose addition.

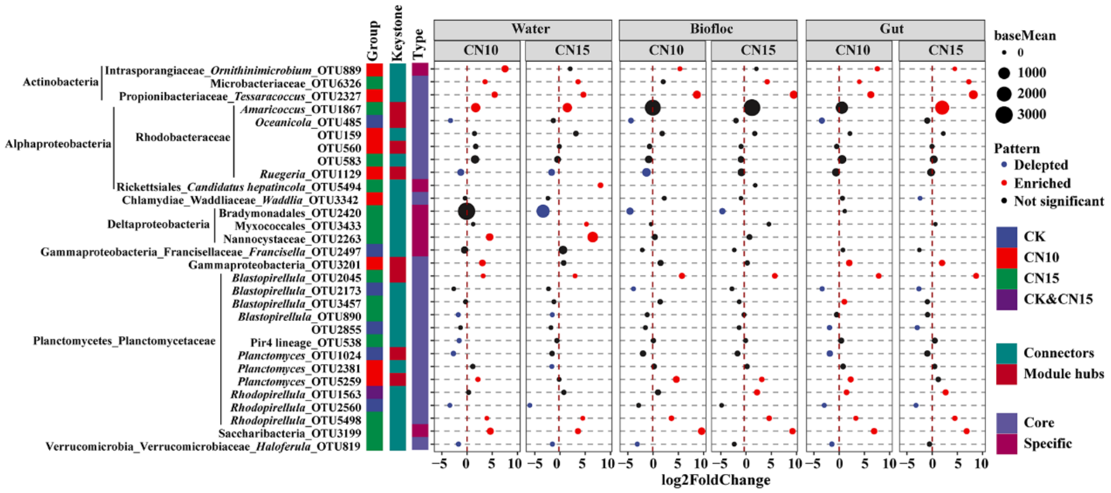

Supplementary Fig. 7 The different degrees of keystone OTUs between control and sucrose addition groups in water, bioflocs and gut according to the DESeq2 analysis. The size of the point represents the average normalized abundance. CK: without sucrose addition; CN10: the C/N ratio of about 10:1 obtained by sucrose addition; CN15: the C/N ratio of about 15:1 obtained by sucrose addition. Each sample had six replicates (n=6), except the water samples (n=4) and biofloc samples (n=2) in control group.

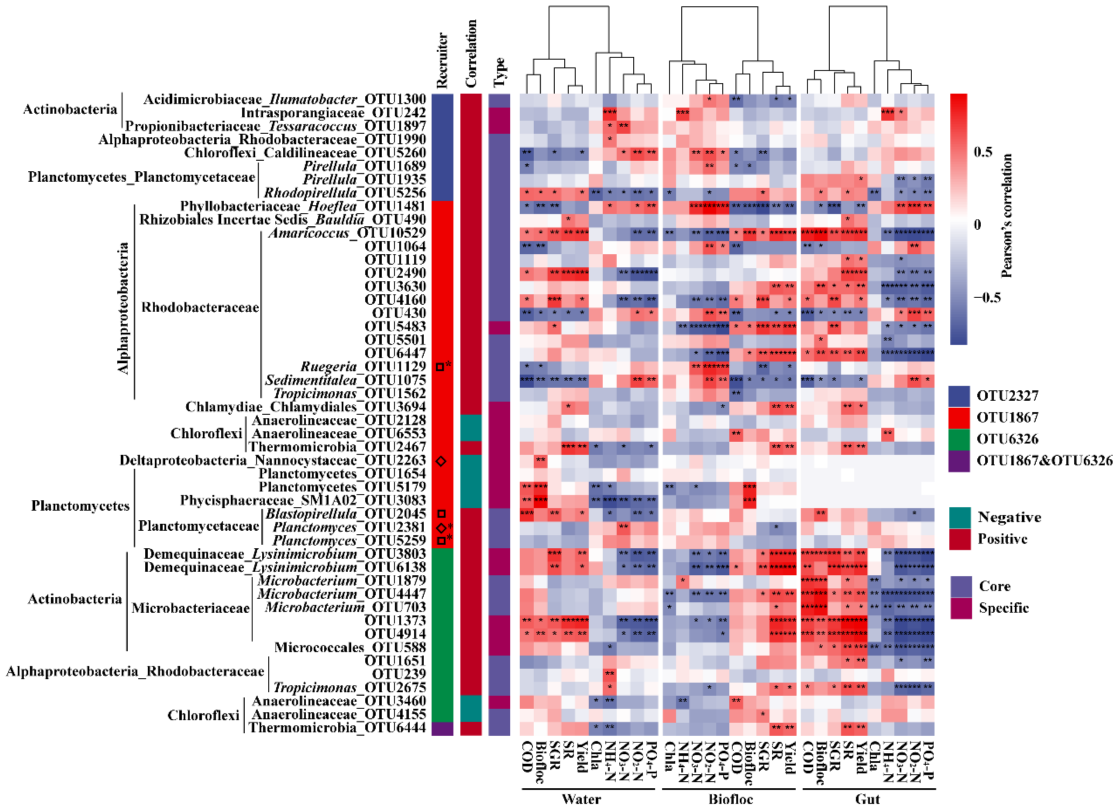

Supplementary Fig. 8 Pearson's correlation analysis between OTUs recruited by three keystone OTUs, water quality parameters, biofloc volume and shrimp growth performance. NH_4^+-N, ammonium-N; NO_2^--N, nitrite-N; NO_3^--N, nitrate- N; PO_4^--P, phosphate-P; COD, chemical oxygen demand; Chl-a, Chlorophyll a; SR, survival rate; SGR, specific growth rate; Yield, total yield of shrimp each pond. Different asterisks indicate a significant difference at *$p < 0.05$, **$p < 0.01$, and ***$p < 0.001$ in C based on Student's t test. CK: without sucrose addition; CN10: the C/N ratio of about 10:1 obtained by sucrose addition; CN15: the C/N ratio of about 15:1 obtained by sucrose addition. Each sample had six replicates (n=6), except the water samples (n=4) and biofloc samples (n=2) in control group.

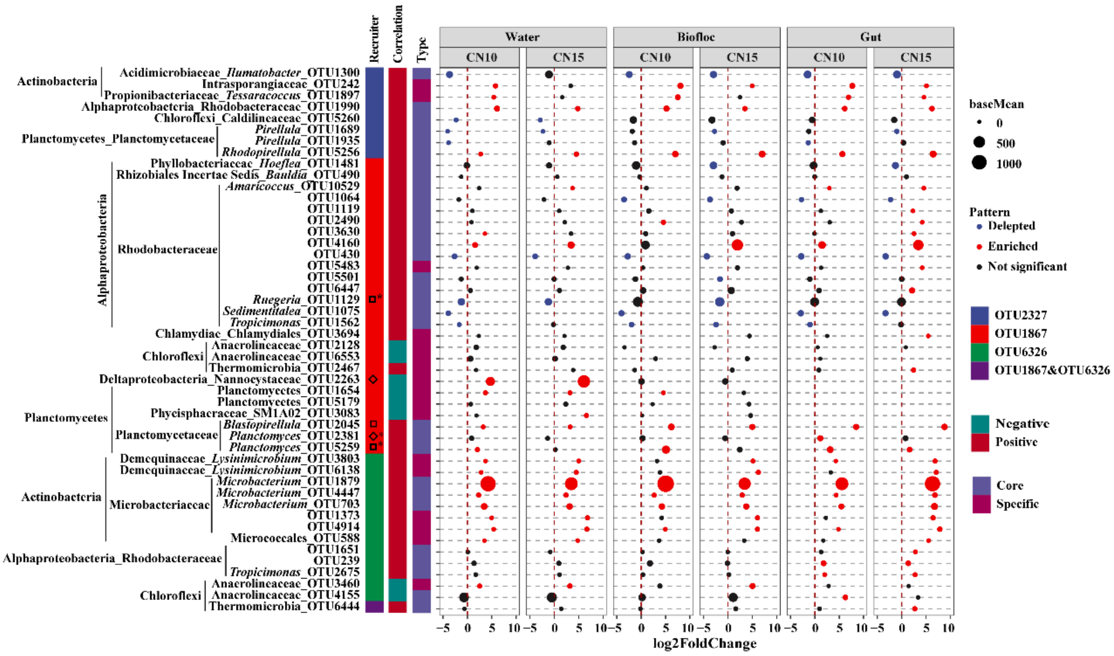

Supplementary Fig. 9 The different degrees of OTUs recruited by three keystone OTUs between control and sucrose addition groups in water, bioflocs and gut according to the DESeq2 analysis. The size of the point represents the average normalized abundance. CK: without sucrose addition; CN10: the C/N ratio of about 10:1 obtained by sucrose addition; CN15: the C/N ratio of about 15:1 obtained by sucrose addition. Each sample had six replicates (n=6), except the water samples (n=4) and biofloc samples (n=2) in control group.

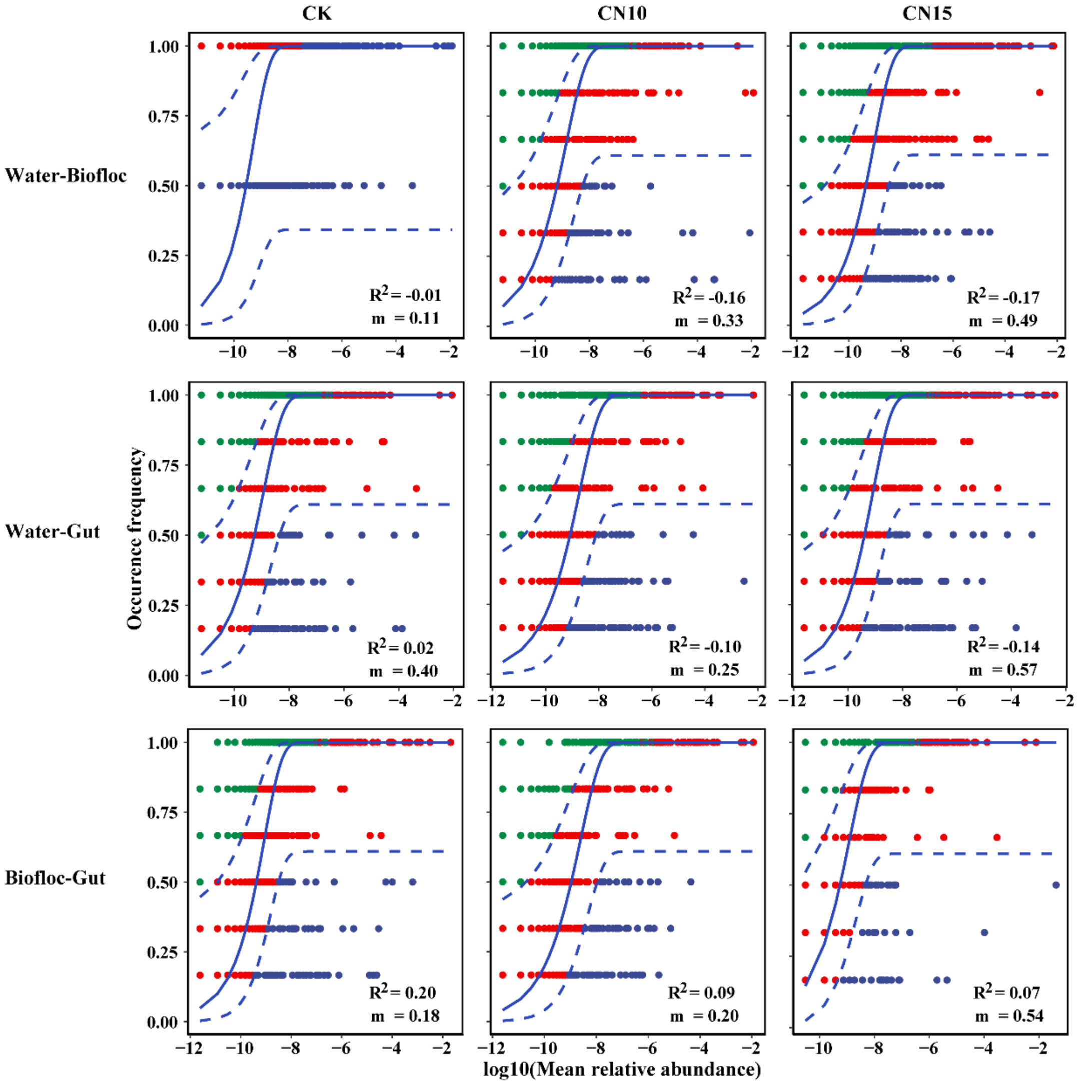

Supplementary Fig. 10 Fit of the neutral models for bacterial communities in bioflocs and gut when the bacterial community in water or bioflocs were used as the bacterial pool. The OTUs that occurred more frequently than predicted by the model are shown in green, while those that occurred less frequently than predicted are shown in blue. Blue dashed lines represent 95% confidence intervals around the model prediction, and the OTUs falling within the confidence intervals were considered neutrally distributed. R^2 values present the goodness of fit of the neutral model and range from 0 (no fit) to 1 (perfect fit). The estimated migration rate (m) was calculated as a measure of dispersal limitation. CK: without sucrose addition; CN10: the C/N ratio of about 10:1 obtained by sucrose addition; CN15: the C/N ratio of about 15:1 obtained by sucrose addition. Each sample had six replicates (n=6), except the water samples (n=4) and biofloc samples (n=2) in control group.

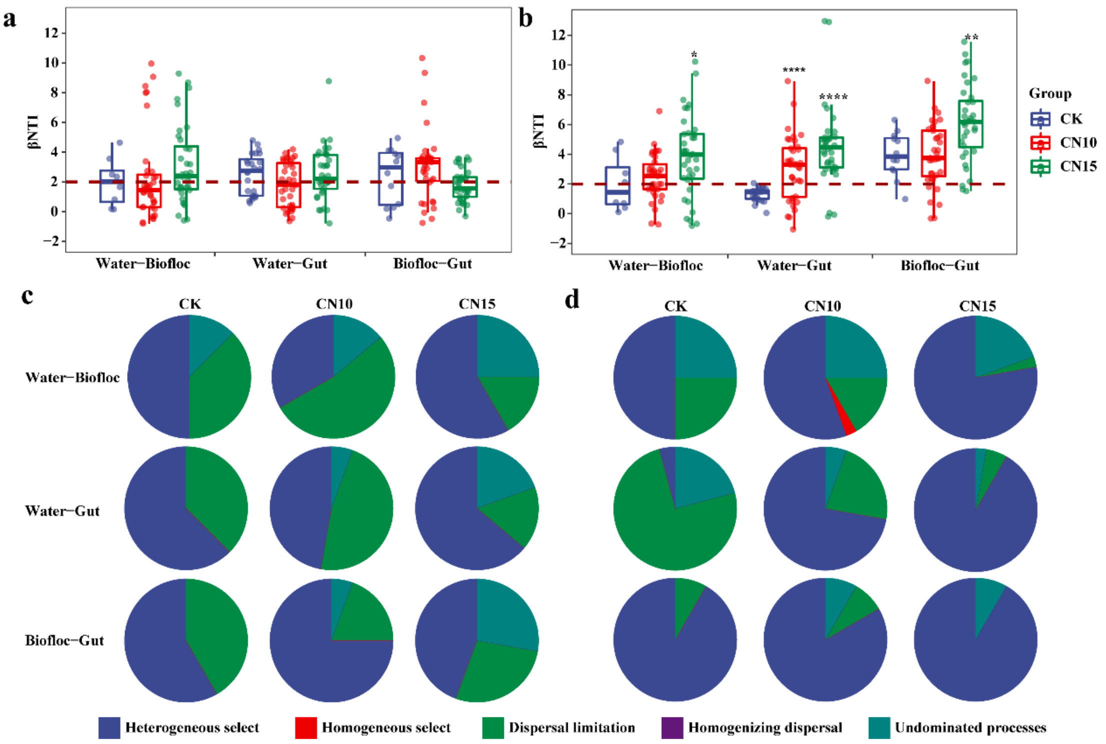

Supplementary Fig. 11 The ecological processes of core and specific taxa from water to bioflocs, water to gut and bioflocs to gut in control and sucrose addition groups. The βNTI values of core **(a)** and specific taxa **(b)**. The boxes represent the median and interquartile range, and whiskers range from minimum to maximum values. Different asterisks indicate a significant difference at $*p < 0.05$, $**p < 0.01$, $***p < 0.001$ and $****p < 0.0001$ based on Student's t test. The contributions of stochastic processes and deterministic process on the core **(c)** and specific taxa **(d)** assembly. CK: without sucrose addition; CN10: the C/N ratio of about 10:1 obtained by sucrose addition; CN15: the C/N ratio of about 15:1 obtained by sucrose addition. Each sample had six replicates (n=6), except the water samples (n=4) and biofloc samples (n=2) in control group.

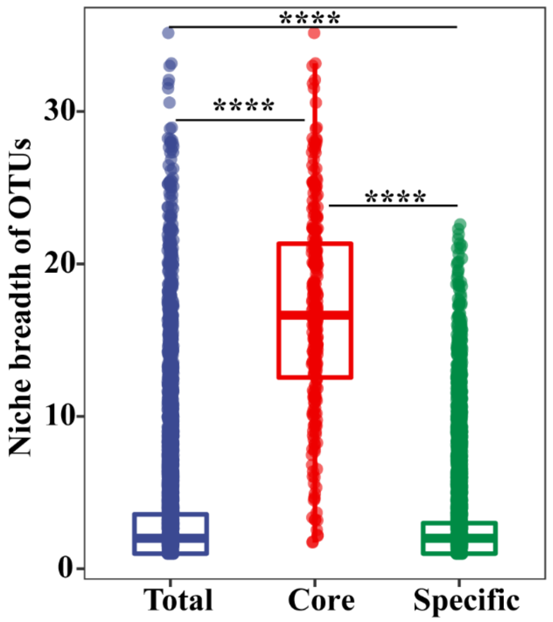

Supplementary Fig. 12 Comparison of mean habitat niche breadth among total, core and specific taxa. The boxes represent the median and interquartile range, and whiskers range from minimum to maximum values. Different asterisks indicate a significant difference at ****$p < 0.0001$ based on Student's t test.
