# Supplementary material for: Sucrose addition directionally enhances bacterial community convergence and network stability of the shrimp culture system
Source: NPJ Biofilms Microbiomes. 2022 Apr 11;8:22. doi: 10.1038/s41522-022-00288-x (PMC9001642; doi:10.1038/s41522-022-00288-x)
Supplement: Supplementary file 2 — Reporting Summary [file 41522_2022_288_MOESM2_ESM.pdf]

nature portfolio

Corresponding author(s): Haipeng Guo

Last updated by author(s): Jan 27, 2022

Reporting Summary

Nature Portfolio wishes to improve the reproducibility of the work that we publish. This form provides structure for consistency and transparency in reporting. For further information on Nature Portfolio policies, see our Editorial Policies and the Editorial Policy Checklist.

Statistics

For all statistical analyses, confirm that the following items are present in the figure legend, table legend, main text, or Methods section.

n/a	Confirmed	
☐	☒	The exact sample size (*n*) for each experimental group/condition, given as a discrete number and unit of measurement
☐	☒	A statement on whether measurements were taken from distinct samples or whether the same sample was measured repeatedly
☐	☒	The statistical test(s) used AND whether they are one- or two-sided<br>*Only common tests should be described solely by name; describe more complex techniques in the Methods section.*
☐	☒	A description of all covariates tested
☐	☒	A description of any assumptions or corrections, such as tests of normality and adjustment for multiple comparisons
☐	☒	A full description of the statistical parameters including central tendency (e.g. means) or other basic estimates (e.g. regression coefficient) AND variation (e.g. standard deviation) or associated estimates of uncertainty (e.g. confidence intervals)
☒	☐	For null hypothesis testing, the test statistic (e.g. *F*, *t*, *r*) with confidence intervals, effect sizes, degrees of freedom and *P* value noted<br>*Give P values as exact values whenever suitable.*
☒	☐	For Bayesian analysis, information on the choice of priors and Markov chain Monte Carlo settings
☒	☐	For hierarchical and complex designs, identification of the appropriate level for tests and full reporting of outcomes
☒	☐	Estimates of effect sizes (e.g. Cohen's *d*, Pearson's *r*), indicating how they were calculated

Our web collection on statistics for biologists contains articles on many of the points above.

Software and code

Policy information about availability of computer code

Data collection	No software was used.
Data analysis	QIIME 1.9.1, R-4.0.3

For manuscripts utilizing custom algorithms or software that are central to the research but not yet described in published literature, software must be made available to editors and reviewers. We strongly encourage code deposition in a community repository (e.g. GitHub). See the Nature Portfolio guidelines for submitting code & software for further information.

Data

Policy information about availability of data

All manuscripts must include a data availability statement. This statement should provide the following information, where applicable:

- Accession codes, unique identifiers, or web links for publicly available datasets
- A description of any restrictions on data availability
- For clinical datasets or third party data, please ensure that the statement adheres to our policy

All sequencing data generated in this study are publicly available from the Genome Sequence Archive in the BIG Data Center, Chinese Academy of Sciences (http://bigd.big.ac.cn/gsa, accession number: CRA005186 and CRA005187)

Field-specific reporting

Please select the one below that is the best fit for your research. If you are not sure, read the appropriate sections before making your selection.

☒ Life sciences ☐ Behavioural & social sciences ☐ Ecological, evolutionary & environmental sciences

For a reference copy of the document with all sections, see nature.com/documents/nr-reporting-summary-flat.pdf

Life sciences study design

All studies must disclose on these points even when the disclosure is negative.

Sample size	A total of 54 samples were collected, including 18 water, 18 bioflocs and 18 gut samples
Data exclusions	Because the DNA concentrations of two water samples and four biofloc samples in control groups were unqualified, a total of 48 DNA samples, including 14 from water, 12 from bioflocs and 18 from gut samples, were used in subsequent experiments.
Replication	Each group or sample has six replications
Randomization	Five shrimps from each tank were randomly selected for sampling.
Blinding	The investigations were blinded.

Reporting for specific materials, systems and methods

We require information from authors about some types of materials, experimental systems and methods used in many studies. Here, indicate whether each material, system or method listed is relevant to your study. If you are not sure if a list item applies to your research, read the appropriate section before selecting a response.

Materials & experimental systems

n/a	Involved in the study	
☒	☐	Antibodies
☒	☐	Eukaryotic cell lines
☒	☐	Palaeontology and archaeology
☐	☒	Animals and other organisms
☒	☐	Human research participants
☒	☐	Clinical data
☒	☐	Dual use research of concern

Methods

n/a	Involved in the study	
☒	☐	ChIP-seq
☒	☐	Flow cytometry
☒	☐	MRI-based neuroimaging

Animals and other organisms

Policy information about studies involving animals; ARRIVE guidelines recommended for reporting animal research

Laboratory animals	The study did not involve laboratory animals.
Wild animals	The study did not involve wild animals.
Field-collected samples	The study did not involve samples collected from field. No ethical approval or guidance was required.
Ethics oversight	No ethical approval or guidance was required because shrimp (Penaeus vannamei) is a kind of food animal.

Note that full information on the approval of the study protocol must also be provided in the manuscript.
